# Developmental Exposure to Kynurenine Affects Zebrafish and Rat Behavior

**DOI:** 10.3390/cells12182224

**Published:** 2023-09-06

**Authors:** Marta Marszalek-Grabska, Kinga Gawel, Nataliia Kosheva, Tomasz Kocki, Waldemar A. Turski

**Affiliations:** Department of Experimental and Clinical Pharmacology, Medical University, Jaczewskiego 8b, 20-090 Lublin, Poland; kinga.gawel@umlub.pl (K.G.); nataliia.kosheva21@gmail.com (N.K.); tomasz.kocki@umlub.pl (T.K.); waldemar.turski@umlub.pl (W.A.T.)

**Keywords:** kynurenine, development, behavior, zebrafish, rat

## Abstract

Proper nutrition and supplementation during pregnancy and breastfeeding are crucial for the development of offspring. Kynurenine (KYN) is the central metabolite of the kynurenine pathway and a direct precursor of other metabolites that possess immunoprotective or neuroactive properties, with the ultimate effect on fetal neurodevelopment. To date, no studies have evaluated the effects of KYN on early embryonic development. Thus, the aim of our study was to determine the effect of incubation of larvae with KYN in different developmental periods on the behavior of 5-day-old zebrafish. Additionally, the effects exerted by KYN administered on embryonic days 1–7 (ED 1–7) on the behavior of adult offspring of rats were elucidated. Our study revealed that the incubation with KYN induced changes in zebrafish behavior, especially when zebrafish embryos or larvae were incubated with KYN from 1 to 72 h post-fertilization (hpf) and from 49 to 72 hpf. KYN administered early during pregnancy induced subtle differences in the neurobehavioral development of adult offspring. Further research is required to understand the mechanism of these changes. The larval zebrafish model can be useful for studying disturbances in early brain development processes and their late behavioral consequences. The zebrafish-medium system may be applicable in monitoring drug metabolism in zebrafish.

## 1. Introduction

Adequate nutrition and reasonable supplementation during pregnancy and breastfeeding are relevant factors for the development of offspring. Among the macro- and micronutrients necessary for fetal growth and development, tryptophan plays an important role. This essential amino acid for protein synthesis is metabolized in five pathways: the kynurenine pathway; the serotonin pathway; the gut microbial pathway; the indole-3-pyruvic acid pathway; and the tryptamine pathway. Many studies have focused on the tryptophan–serotonin pathway, indicating the protective effect of tryptophan supplementation. However, 95% of dietary tryptophan is metabolized through the kynurenine pathway [1]. The initial and rate-limiting step of the kynurenine pathway is the oxidation of tryptophan to *N*-formyl-kynurenine [2,3]. Most of tryptophan is oxidized by tryptophan 2,3-dioxygenase (TDO) in liver cells. In other types of cells, tryptophan can be metabolized by an inducible enzyme, i.e., indoleamine 2,3-dioxygenase (IDO), which is transcribed under certain pathophysiological conditions, including stress and inflammation [4]. Kynurenine (KYN) is the central metabolite of the kynurenine pathway and a direct precursor of kynurenic acid (KYNA), anthranilic acid, and 3-hydroxykynurenine, as well as other downstream metabolites formed along the kynurenine pathway [5]. The kynurenine pathway metabolites possess immunoprotective or neuroactive properties, with the ultimate effect on fetal neurodevelopment [6,7]. It has been postulated that increased levels of the kynurenine pathway metabolites are implicated in the pathogenic mechanisms that contribute to aberrant neurodevelopment, which precedes the clinical onset of schizophrenia and bipolar disorder [8,9,10]. This hypothesis was built upon the fact that KYNA is a naturally occurring astrocyte-derived antagonist of *N*-methyl-d-aspartate (NMDA) [11,12,13] and conceivably the alpha-7 nicotinic receptor (α7nAChR) [14]. KYNA is a metabolite in the neuroprotective branch of the kynurenine pathway, and it is suggested that increased concentration of KYNA causes alterations in glutamatergic and cholinergic, and indirectly, in dopaminergic signaling, hereby leading to symptoms of schizophrenia. On the other hand, quinolinic acid, largely synthesized in microglia, is an NMDA receptor agonist [11]. It can generate free radicals and contribute to neurotoxicity. In addition, increased levels of 3-hydroxykynurenine and anthranilic acid have been reported in patients with schizophrenia [15].

KYNA also acts as a ligand of G protein-coupled receptor 35 (GPR35) and the aryl hydrocarbon receptor (AhR), two signaling receptors that are functional in both the brain and peripheral organs [16,17]. AhR regulates the functions of a plethora of cells in both the innate and adaptive immune systems, including inflammation and carcinogenesis processes [18,19,20,21]. It should, however, be noted that AhR is an intracellular receptor, and so far, no uptake of KYNA has been demonstrated. Thus, it is uncertain whether exogenously administered KYNA can effectively influence this receptor [22]. However, recent data indicated that tryptophan depletion strongly increases AhR expression, potentiating its activation by weak agonists such as KYN and its derivatives [23]. Furthermore, tryptophan depletion also increased cellular KYN uptake by increasing SLC7A5 (LAT1) expression, thereby also contributing to potentiating the capacity of KYN to activate the AhR on tryptophan depletion.

KYN is able to cross the placenta and the fetal blood–brain barrier as a single oral administration of KYN to pregnant mouse dams has been found to raise the KYN levels in the fetal plasma and brain [24]. In studies conducted on rats, continuous KYN supplementation in wet mash to pregnant dams during the last week of gestation has been found to result in elevated levels of maternal and fetal plasma KYN and increased fetal KYNA levels [25]. Furthermore, KYN administration during pregnancy induced memory impairments in adult offspring as well as disrupted sleep behavior [25,26,27,28,29,30,31]. In our previous study, intragastric supplementation of KYN to pups from birth to weaning resulted in neurobehavioral changes in adult offspring with a greater pronunciation in male than in female rats [32].

In addition to the effect of KYN supplementation during pregnancy, a number of studies on the modification of the kynurenine pathway during pregnancy and breastfeeding have been conducted [33,34,35,36]. It was shown that inhibition of the kynurenine pathway in late gestation induced molecular and functional changes in the central nervous system of the offspring, suggesting a significant role of this pathway in the development of the embryonic and neonatal nervous system.

**Figure 1:**
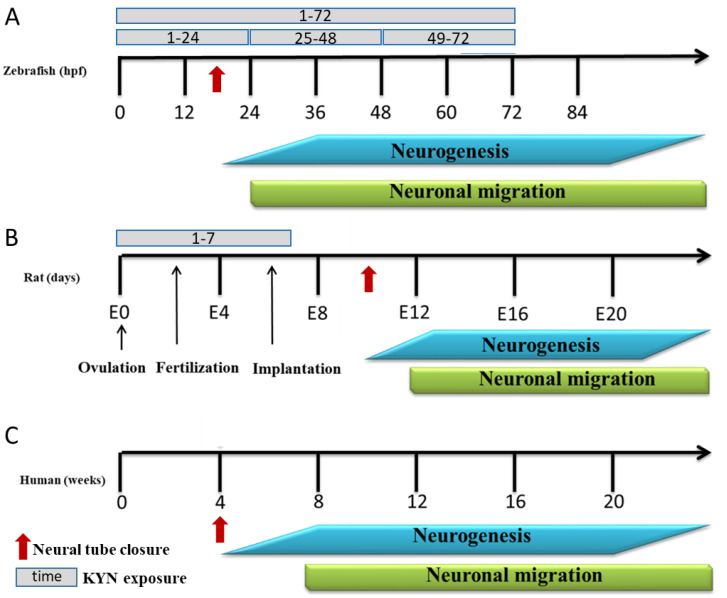
Schematic representation of the embryonic brain development timing in zebrafish (**A**), rats (**B**), and humans (**C**). Following the neural tube closure (arrows), embryonic brain development implicates three major processes: proliferation of progenitor populations; lineage decisions; and migration. Data adapted from [39,52]. Hpf—hours post-fertilization; KYN—kynurenine.

Although numerous studies focusing on KYN supplementation during late pregnancy and breastfeeding have been conducted, knowledge about the influence of KYN on early embryonic development is still negligible. After fertilization, the zygote begins the process of early embryonic development in which it undergoes several rounds of cleavage to increase cell number and then forms a blastocyst, while at the same time, the embryo gets rid of both maternal and paternal influences to start its own life with its own genetic material. Early embryonic development is a tightly controlled process that is conserved across mammalian species, although details vary from species to species [37]. The first major event of the central nervous system development in all vertebrates is the formation of a specialized fold of ectodermal tissue called the neural tube, from which the spinal cord and the brain are subsequently differentiated. The neural tube formation occurs in rats approximately in mid-gestation, on gestational days 10.5–11, in humans between 3 and 4 weeks, and in zebrafish between 12 and 24 h post-fertilization (hpf), respectively (Figure 1) [38,39,40,41]. Neural development interruptions during this early period can result in severe abnormalities of the brain and the spinal cord. Extreme cases of spina bifida (“divided spine”) may lead to failure in the closure of the neural tube and severe defects in either the spinal cord or the brain [39].

To investigate the potential neurodevelopmental toxicity of the kynurenine pathway, an in vivo model is needed to assess the effects exerted by the kynurenine pathway metabolites on developmental changes over short periods. The zebrafish (*Danio rerio*) model is rapidly becoming a useful tool in toxicology studies and seems appropriate for this type of research. The factors that make zebrafish an attractive research model are its simple breeding and maintenance requirements, high fecundity, rapid external development, larval body transparency, and the presence of almost all human-like internal organs [42]. Zebrafish share high physiological and genetic homology with humans, with over 70% of disease-associated genes in humans having identifiable orthologs in zebrafish [42,43]. Zebrafish have similar brain architecture to humans. Most brain structures are presented in zebrafish [44]; a blood–brain barrier develops on day 3 post-fertilization (dpf) and becomes fully functional by 10 dpf, sharing structural and functional similarities with mammals. Before 10 dpf, the blood–brain barrier is leaky, allowing the smaller-sized molecules to pass through [45,46]. Therefore, it is possible to have drugs delivered through a simple incubation of embryos and larvae in a medium solution.

To date, no studies have evaluated the effects of KYN embryonic exposure on larval zebrafish behavior. Thus, we aimed to assess the effect of incubation with KYN in different developmental periods on the behavior of 5-day-old larval zebrafish. Moreover, we analyzed the fate of KYN in the zebrafish medium in order to determine whether zebrafish were able to absorb KYN from the medium and metabolize it. Additionally, we tested the effects exerted by KYN administered on embryonic days 1–7 (ED 1–7) on the behavior of adult offspring of rats.

## 2. Materials and Methods

### 2.1. Zebrafish

Zebrafish (*Danio rerio*) embryos of the AB strain were purchased from the Experimental Medicine Centre, Medical University of Lublin (Lublin, Poland). Embryos and larvae up to 120 hpf were kept under generally accepted environmental conditions, i.e., in incubators at 28.5 °C, with 14/10 h light/dark cycles. They were reared in Danieau’s buffer, i.e., in an embryo medium (1.5 mM Hepes, pH 7.6, 17.4 mM NaCl, 0.21 mM KCl, 0.12 mM MgSO_4_, and 0.18 mM Ca(NO_3_)_2_). Ethical permission is not required for experiments with embryos and larval zebrafish up to 120 hpf. Nevertheless, all efforts were made to minimize animals’ suffering and to reduce to the minimum the number of larvae used. Tricaine (15 μM) was used for euthanasia immediately after the experiments.

### 2.2. Rats

Twelve pregnant Wistar dams were obtained from the Experimental Medicine Centre, Medical University of Lublin (Lublin, Poland). After birth, the offspring stayed with their mothers. To avoid potential competition between males and females in access to the mother’s milk, on postnatal day (PND2), the animals were assigned to mothers who fed individuals of only one sex. The pups were weaned on PND21 and group-housed according to gender. Rats of both sexes in gender-segregated groups were used in the experiments. The animals were maintained under standard laboratory conditions (12 h light/dark cycle, room temperature 21 ± 1 °C) with free access to tap water and laboratory chow (Sniff Spezialdiäten GmbH, Soest, Germany). Each experimental group was composed of 8–10 animals. All the experiments were conducted according to the National Institute of Health Guidelines for the Care and Use of Laboratory Animals and the European Community Council Directive for the Care and Use of Laboratory Animals of 22 September 2010 (2010/63/EU). This study was approved by the Local Ethics Committee for Animal Experiments, Lublin, Poland (No. 127/2022).

### 2.3. Chemicals

L-kynurenine sulfate (KYN), D-amphetamine sulfate, and dizocilpine (5-methyl-10,11-dihydro-5H-dibenzo [a,d] cyclohepten-5,10-imine) were obtained from Sigma-Aldrich, Saint Louis, MO, USA. In the zebrafish larvae experiments, KYN was dissolved in the embryo medium. For rat studies, all drugs were dissolved in saline and prepared *ex tempore*. The pH of the KYN solution was adjusted to 7.0 with 0.1 N NaOH. KYN was administered intragastrically at a dose of 50 mg/kg in a volume of 5 mL/kg to 6 of the 12 dams on ED 1–7. The control group received a saline solution (Figure 1). D-amphetamine sulfate (1.0 mg/kg) and dizocilpine (at doses of 0.075, 0.15, and 0.3 mg/kg) were administered intraperitoneally (i.p.) in a volume of 2 mL/kg. In all the cases, the control animals received the corresponding saline injections. High-performance liquid chromatography (HPLC) reagents were purchased from J.T. Baker Chemicals (Gliwice, Poland) or Sigma-Aldrich (Saint Louis, MO, USA).

### 2.4. Biochemical Analysis

#### 2.4.1. Tissue Sampling and Preparation

Plasma was collected from mothers and young offspring after breastfeeding and from adult offspring. The animals were decapitated, and the plasma was obtained by centrifugation of the blood at 1620× *g* for 30 min (temp. 4 °C). The samples were stored at −80 °C until further processing. The blood plasma was deproteinated with 8% perchloric acid. The samples were vortexed, kept at 4 °C for 10 min, centrifuged at 20,598× *g* for 30 min at 4 °C, and prepared for analysis using HPLC.

#### 2.4.2. Determination of Tryptophan, KYN, and KYNA in Animal Plasma

Tryptophan, KYN, and KYNA concentrations were measured according to the method of Zhao et al. [47]. In brief, the studied substances were analyzed by the HPLC system (The UltiMate 3000 Analytical systems (Thermo Fisher Scientific, Waltham, MA, USA)). The samples were separated on an analytical column (Agilent HC-C18; 250 × 4.6 mm, inner diameter). The mobile phase was composed of 20 mmol/L NaAc, 3 mmol/L ZnAc_2_, and 7% acetonitrile. It was pumped at a flow rate of 1 mL/min, and the volume per injection was 100 μL. The wavelength of the UV detector was set at 250 nm for tryptophan, 365 nm for KYN, and the RF 2000 Fluorescence Detector (Dionex) was set at an excitation wavelength of 348 nm and emission at 398 nm for KYNA determination. Chromeleon 7.2 software was used to control the HPLC system and record chromatographic data.

#### 2.4.3. Determination of KYN and KYNA in Zebrafish Medium

The content of KYN and KYNA in the zebrafish medium was measured. Zebrafish larvae were incubated in the highest concentrations of KYN (100 μM, established as a maximum tolerated concentration (MTC)) from 72 to 119 hpf. Medium was exchanged every 24 h and frozen until the analysis. Next, the contents of KYN and KYNA were measured as described above.

### 2.5. Behavioral Tests in Zebrafish

#### 2.5.1. Determination of the Maximum Tolerated Concentration

MTC assay had been performed before the behavioral experiments took place in order to determine the non-toxic KYN concentration. To this end, zebrafish embryos were screened an hour after fertilization, and only fertilized and completely transparent embryos were transferred to 12-well plates. In each well, at least 20 embryos (twice replicated) were kept in 3000 μL of the medium, either without or supplemented with increasing concentrations of KYN (from 10 to 200 μM). Then, they were incubated at 28.5 °C at 1–96 hpf. The KYN solutions were replaced every 24 h with fresh ones. In 96 hpf old zebrafish, the following parameters were scored: posture; body length; heart/yolk edema; signs of yolk necrosis; swim bladder inflation; heartbeat; and jaw malformation. Additionally, their response to touch was scored in order to choose the dose that had no negative impact on muscle performance or function [48]. The highest concentration of KYN (i.e., 100 μM) that did not induce any visible signs of toxicity and did not affect touch response was chosen as MTC for behavioral experiments [48,49,50].

#### 2.5.2. Locomotor Activity 

Zebrafish embryos or larvae were incubated in two concentrations of KYN, 10 μM and 100 μM, at different time points, i.e., one cohort was incubated from 1 to 72 hpf (from cleavage to the hatching period), the second from 1 to 24 hpf (from cleavage to the segmentation period), the third from 25 to 48 hpf (the pharyngula period), and the last from 49 to 72 hpf (the hatching period) (Figure 1) [51]. When the incubation time elapsed, the embryos were thoroughly washed at least 5 times in order to completely get rid of KYN. After that, they were incubated in a zebrafish medium until the behavioral analysis, with medium exchange every 24 h. The locomotor activity of the larvae was assessed at 119 hpf in 50% light. Briefly, the larvae were placed individually in the wells of a 48-well plate filled with 300 μL of the medium solution (individual larva per well). Subsequently, the plates were positioned in an automated tracking device (Noldus, Wageningen, Netherlands), and the larvae were habituated for 10 min at 28.5 °C. Then, the plates with the larvae were tracked for 30 min at 5 min integration intervals. All measurements were performed during the same daytime period. The distance (in millimeters) covered by each larva was recorded.

#### 2.5.3. Light–Dark Transition Assay 

The zebrafish embryos or larvae were incubated in two concentrations of KYN: 10 μM and 100 μM as described above. The light–dark transition assay was conducted at 119 hpf. For this, each larva was placed individually in the wells of a 48-well plate filled with 300 μL of the medium solution. Subsequently, the plates were positioned in an automated tracking device (Noldus, Wageningen, The Netherlands), and the larvae were accustomed to environmental conditions for 10 min at 28.5 °C. Then, the plate larvae were tracked for 20 min at 5 min integration intervals, in the following conditions: the 100% light phase for 10 min and then the dark phase for 10 min [53]. All measurements were performed in the same daytime period. The distance (in millimeters) covered by each larva was recorded.

### 2.6. Behavioral Tests in Rats

Behavioral tests were performed in two separate cycles on two different groups of rats aged 56, 63, and 70 days, respectively. There was a 7-day interval between individual tests. The behavioral tests were carried out according to the following principle: from the least to the most invasive, in order to minimize the risk of a possible negative impact of the test conditions/procedure on the results obtained in the subsequent test.

During the first cycle of the behavioral experiments, Wistar rats were subjected to a spontaneous locomotor activity test on day 56, an elevated plus-maze test on day 63, and an amphetamine-induced locomotor activity test on day 70 of the animals’ age.

During the second cycle of the behavioral experiments, Wistar rats were subjected to a dizocilpine-induced locomotor activity test on day 56, a Barnes maze test on day 63, and a contextual and cued fear conditioning test on day 70 of the animals’ age.

A detailed description of these behavioral tests can be found in our previous paper [32].

### 2.7. Statistical Analysis

The data that followed Gaussian distribution were assessed using two-way ANOVA to evaluate the significance of differences between the groups, and Tukey’s test for multiple comparisons was used as a post hoc analysis if any significant differences were found. The data with non-Gaussian distribution were evaluated using the Kruskal–Wallis test (non-parametric ANOVA) followed by post hoc Dunn’s multiple comparisons test if a significant difference was found or by a Chi-square test. Student’s *t*-test was used if applicable.

The multi-way contingency table Chi-square test was used to conduct mobility analysis based on locomotor activity categories. In this analysis, the larvae were classified into one of the following categories: low activity (lower 95% CI); intermediate activity, and high activity (upper 95% CI). Confidence limits (CL) were calculated for the control group and were applied to the groups exposed to both doses of KYN.

The differences were considered statistically significant if the *p*-value was lower than 0.05. Graphs were generated using Prism 8 GraphPad (GraphPad Software Inc., San Diego, CA, USA).

## 3. Results

### 3.1. The Effect of Incubation with KYN at Different Periods on the Behavior of 5-Day-Old Larval Zebrafish

#### 3.1.1. Locomotor Activity in Zebrafish

**Figure 2:**
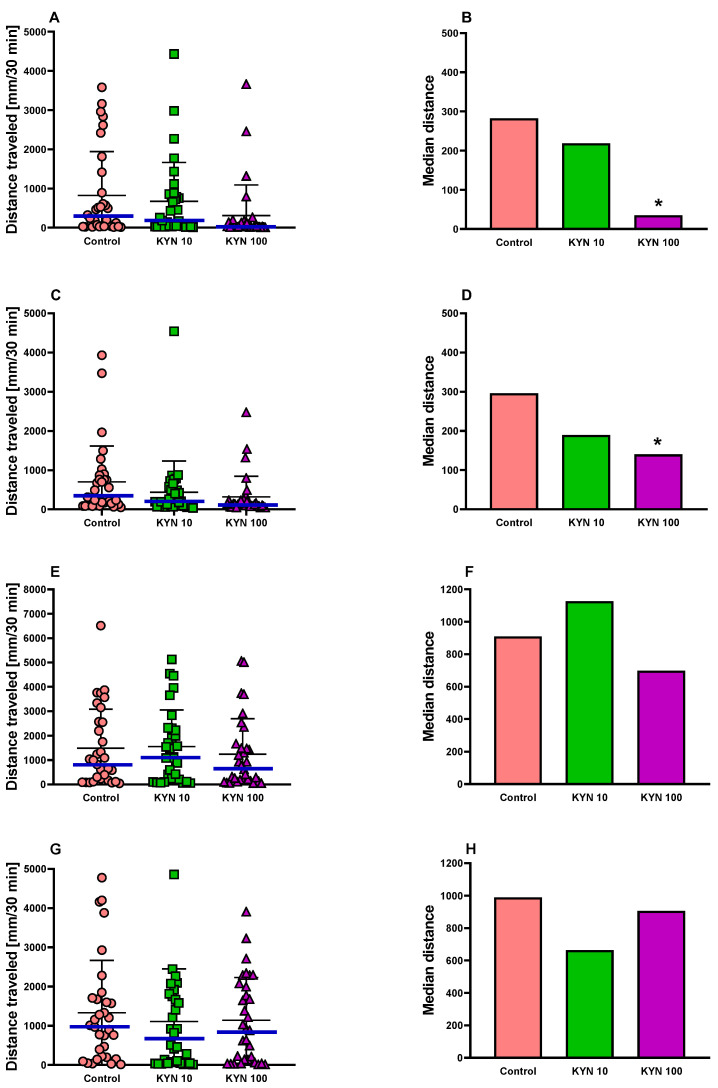
Locomotor activity of 5-day-old larval zebrafish exposed to KYN from 1 to 72 (**A**,**B**), from 49 to 72 (**C**,**D**), from 25 to 48 (**E**,**F**), and from 1 to 24 (**G**,**H**) hpf. Distance traveled for 30 min by each larva (**A**,**C**,**E**,**G**). Mean ± standard deviation (SD) plotted in black. The median value displayed as a horizontal bar in blue. Median distance traveled for 30 min, calculated for each group (**B**,**D**,**F**,**H**). Number of subjects = 31–32 per group. * *p* < 0.05 vs. controls, non-parametric ANOVA. KYN—kynurenine. Note that the vertical axis is different on the left and right panels.

The behavior of 5-day-old larval zebrafish exposed to KYN at different periods was assessed by the locomotor activity test. The non-parametric Kruskal–Wallis test showed that there was a statistical difference in the distance traveled by the zebrafish incubated with KYN from 1 to 72 hpf (*p* = 0.0074) and from 49 to 72 hpf (*p* = 0.014). Dunn’s multiple comparisons test indicated that the zebrafish incubated with KYN at a dose of 100 μM displayed reduced basal locomotor activity compared to the control group (*p* < 0.05, Figure 2).

**Figure 3:**
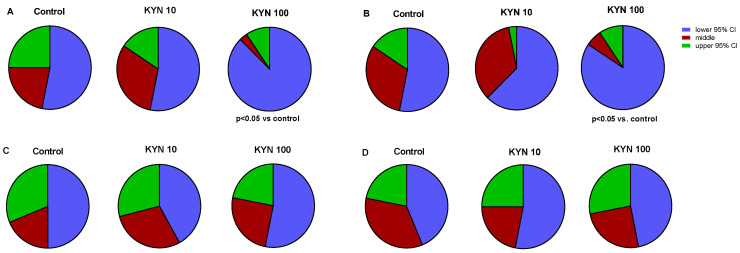
Locomotor activity of 5-day-old larval zebrafish exposed to KYN from 1 to 72 (**A**), from 49 to 72 (**B**), from 25 to 48 (**C**), and from 1 to 24 (**D**) hpf. Data are presented as a proportional pie graph. Both 95% lower and upper confidence intervals (95% CI) were calculated for the control group and were applied to the groups exposed to both doses of KYN. Consequently, each larva was classified into one of the following categories: low activity (lower 95% CI); intermediate activity; and high activity (upper 95% CI). The obtained data were subjected to multi-way contingency table Chi-square analysis. The *p*-value of <0.05 was considered significant. Number of subjects = 31–32 per group. KYN—kynurenine.

A Chi-square test was performed to examine the mobility pattern in the larvae population. Statistically significant differences were observed in the zebrafish incubated with KYN from 1 to 72 hpf (Chi^2^ = 9.46) and from 49 to 72 hpf (Chi^2^ = 8.11) (*p* < 0.05, Figure 3). It was evidenced that among the larvae incubated with KYN at a dose of 100 μM, the number of individuals in the lower 95% CI category distinctly increased at the expense of the larvae with higher motor activity, indicating a decrease in larval mobility. The incubation of larvae with KYN from 25 to 48 hpf and from 1 to 24 hpf did not affect the basal locomotor activity of zebrafish (Figure 2 and Figure 3).

#### 3.1.2. Light–Dark Transition Assay in Zebrafish

Since incubation with KYN from 1 to 72 hpf and from 49 to 72 hpf resulted in changes in the basal locomotor activity, the effect of KYN exposure during these time intervals on the light–dark transition test was assessed. The conducted experiments showed no changes in zebrafish behavior in either the light phase or the dark phase, respectively (Figure S1).

#### 3.1.3. Determination of KYN and KYNA in Zebrafish Medium

**Figure 4:**
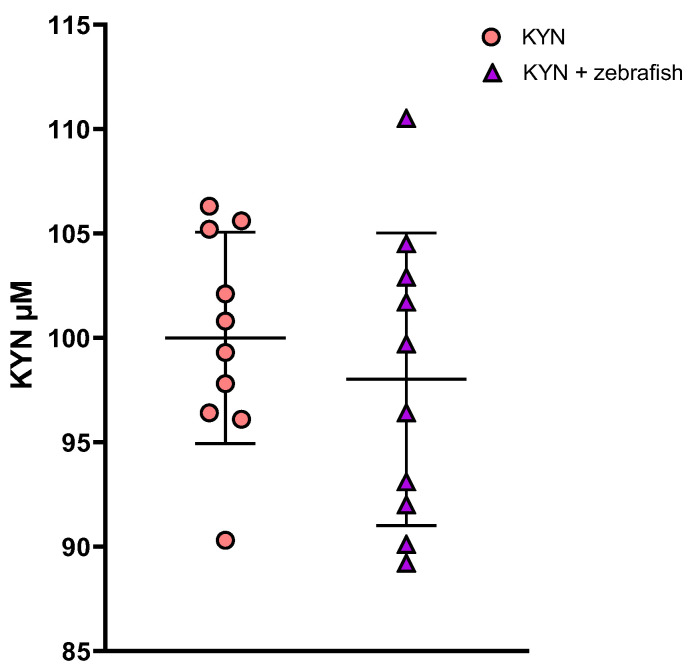
The content of KYN in the zebrafish medium inhabited by larvae at 96 to 119 hpf. Data are presented as a mean ± standard deviation (SD); circles—medium without larvae; triangles—medium inhabited by larvae (20 larvae per well). Number of wells = 10 per group. KYN—kynurenine.

Larvae aged 72–119 hpf did not affect the KYN content in the medium. The depletion of KYN did not exceed 2% and did not reach statistical significance in any of the 24-hour periods studied. A representative result is shown in Figure 4. However, a detailed analysis of the chromatograms revealed at least three remarkable peaks that appeared only in the medium after 24-hour incubation with larvae (96–119 dpf) (Figure S2).

A peak in retention time corresponding to the KYNA standard was observed in the medium after 24-hour incubation with larvae (96–119 dpf). The magnitude of the peak lay only slightly above the detection limit but was distinctly higher than in medium without larvae (Figure S3). The estimated amount of KYNA production under these conditions was 4.23 ± 1.02 pmol/20 larvae/24 h. The addition of an internal standard containing authentic KYNA to the original sample indicated the same retention time (Figure S4).

### 3.2. The Effect of KYN on the Level of Tryptophan, KYN and KYNA in Rat Plasma

The intragastric administration of KYN to pregnant dams on ED 1–7 increased levels of tryptophan, KYN, and KYNA in blood plasma of mothers after breastfeeding. In young males, the level of tryptophan was reduced, the level of KYN was increased in both sexes, and the level of KYNA was decreased in male rats. In adult offspring, tryptophan level was reduced in both sexes, whereas the levels of KYN and KYNA were unchanged (Table 1 and Tables S1–S3).

### 3.3. The Effect of KYN on the Body Mass Gain of Young and Adult Rats

The intragastric administration of KYN to pregnant dams on ED 1–7 decreased the body mass gain in male offspring rats on PND 8, 12, 20, and 35 and in female offspring rats on PND 12–63 (Figure S5).

### 3.4. The Effect of KYN on the Locomotor Activity of Adult Rats

To assess the effect of intragastric administration of KYN to pregnant dams on ED 1–7 on the behavior of adult offspring (56-day-old) rats, various parameters of locomotor activity were analyzed. It was found that KYN administration on ED 1–7 did not affect the spontaneous locomotor activity of adult offspring male rats (Table 2, Figure S6) but reduced the vertical activity of adult female offspring rats (Table 3, Figure S7). To further investigate the susceptibility of adult offspring rats to stimulants, amphetamine and dizocilpine were administered immediately before the locomotor activity test. It was established that KYN administration on ED 1–7 reduced the susceptibility of adult offspring male and female rats to the action of dizocilpine and had little effect on the action of amphetamine (Table 2 and Table 3, Figures S6 and S7).

### 3.5. The Effect of KYN on the Anxiety-like Behavior of Adult Rats

KYN administration on ED 1–7 did not change the number of entries into the open arms, the time spent by the rats in the open arms, and the number of total entries into both the open and closed arms in adult offspring of both sexes (Tables S4 and S5, Figure S8). Thus, the treatment with KYN on ED 1–7 had no effect on the anxiety-like behavior as compared to the respective control groups.

### 3.6. The Effect of KYN on the Spatial Memory and Associative Learning of Adult Rats

A reversal learning experiment in the Barnes maze was conducted 24 h after completing the training, which lasted three consecutive acquisition days. Adult male offspring attempted more entries into the previous escape location (*p* < 0.05 by Student’s *t*-test) (Table S4, Figure S9). Adult female offspring did not differ from their respective control counterparts (Table S5, Figure S9).

There were no significant differences during the training of the fear conditioning test, the contextual fear conditioning test, and the cue fear conditioning test between adult offspring of both sexes and their control counterparts (Tables S4 and S5, Figure S10).

## 4. Discussion

The present study is the first to evaluate behavioral consequences of the transient KYN exposure of embryos and larval zebrafish during neurodevelopmental periods. Our study revealed that the exposure of zebrafish to KYN in the concentrations tested (i.e., 10 and 100 μM) did not induce any visible signs of toxicity. However, the locomotor activity test results indicated some behavioral disturbances in zebrafish larvae exposed to KYN. Particularly significant changes in zebrafish behavior were noticed when zebrafish embryos or larvae were incubated in the medium with KYN at a concentration of 100 μM from 1 to 72 hpf and from 49 to 72 hpf. However, a similar trend could also be seen at a dose of 10 μM. To test whether an analogous effect would occur in rodents, we performed a corresponding study on rats and also noted subtle changes in the behavior of adult animals.

Our research revealed that the incubation of embryos and larvae with KYN from 1 to 72 hpf and from 49 to 72 hpf reduced the distance traveled in the basal locomotor activity measured under standard conditions, which is an important factor that can determine the optimal navigation of fish in the environment [54]. Surprisingly, KYN-treated larvae responses in the light–dark transition test during this period of development, i.e., 1–72 hpf and 49–72 hpf, were not affected. The light–dark transition assay allows us to assess the anxiety and stress behavior of the larvae toward sudden changes in light, in particular, a substantial increase in activity upon a switch to darkness [55]. This test is believed to reflect the nervous system, visual pathways development, and the integrity of the brain function, which renders it particularly sensitive to neuroactive and neurotoxic agents [56,57,58]. In the dark phase of this assay, compounds with anxiolytic properties decrease, whereas compounds with anxiogenic properties increase the distance traveled by zebrafish [59,60,61]. This was, however, not the case in our experiments since we did not observe any differences among the tested groups of animals in the dark phase.

KYN is a well-known agonist of the AhR receptor [18]. AhR receptors are ligand-dependent transcription factors that translocate from the cytoplasm to the nucleus upon ligand binding, where they dimerize with the AhR nuclear translocator protein (ARNT). Subsequently, this heterodimer binds to a specific DNA sequence, the so-called dioxin response element, to induce transcription of a number of target genes, e.g., *cyp1a*, encoding cytochrome P450 1 A (CYP1A) [62]. Unlike the singular form of AhR in humans, zebrafish are found to possess three AhR genes: *ahr1a*; *ahr1b* (paralogs); and *ahr2* [63,64]. Although *ahr1a* is the ortholog of human AhR in zebrafish, *ahr2* is also functional in this species [65,66]. Additionally, zebrafish also have two *arnt* genes with three splice variants each (*arnt1a/b/c* and *arnt2a/b/c*). During zebrafish development, *ahr1a* expression is detected from 24 hpf, and its expression increases up to 72 hpf, being fairly even up to 120 hpf. Similar to *ahr1a*, *ahr1b* expression is detected from 24 hpf and continues to rise to 48 hpf [64]. Recently, Sugden et al. [67] showed that *ahr1a* and *ahr1b* displayed restricted expression to the liver and the eye, respectively. Interestingly, *ahr1b* was necessary to keep the basal expression of *cyp1a1* in hindbrain vasculature in the absence of *ahr2*. The authors concluded that, in a situation where one of the genes is absent, some other forms might compensate for its activity [67]. Moreover, *ahr2* itself is expressed earlier than other AhR genes. Andreassen et al. [64] showed that *ahr2* was evenly expressed in a 12-hpf-old embryo, whereas its expression at 24 hpf was observed in some brain regions like the optic tectum and the cerebellum. It overlapped with the expression of *arnt2*. At 120 hpf, the expression of both *ahr2a* and *arnt2* in the larvae brain was no longer visible [64]. Only recently, it was shown that larval zebrafish with *ahr2* deficiency have impaired behavioral responses (hyperactivity) [68].

The mutual interaction of all these genes and the bunch of their downstream targets in zebrafish, in both physiological and toxicological circumstances, is very complex [67,69,70,71,72] and yet to be explored despite very extensive work performed (for a recent comprehensive review, see 66). Incardona et al. [73] pointed out that, taking into account the complexity of AhR-related mechanisms in zebrafish, it is not possible to predict a ligand’s toxicity based only on its potency. In Incardona’s paper, the authors chronically incubated zebrafish embryos and larvae in different AhR ligands-polycyclic aromatic hydrocarbons (PAHs) (pyrene, chrysene, benz[*a*]anthracene, and benz[*b*]anthracene) and analyzed the morphological descriptors as a readout of toxicity. Even though they used compounds from the same chemical group, they found completely different phenotypes in zebrafish larvae, from mild to moderate to severe [73]. Among all the AhR ligands tested in zebrafish, 2,3,7,8-tetrachlorodibenzo-*p*-dioxin (dioxin, TCDD) is the most potent, and it has been extensively studied in the zebrafish model [74]. It was evidenced that zebrafish treated with TCDD at a very early stage of development (i.e., 6 hpf, for 1 h) exhibited cardiovascular dysfunction, impaired osmoregulation, anemia, hemorrhage, skeletal malformations, growth retardation, non-inflated swim bladder, and post-hatch mortality. However, the TCDD-related toxicity was delayed and did not manifest until 48–120 hpf, which is when the morphogenesis of the primary organ systems and embryo growth occurs [75]. The authors have also observed altered brain morphology, but they did not analyze larval behavior [75]. Only recently, Martin et al. [76] showed that early exposure to TCDD disrupted embryonic brain morphology, and they found out that the appropriate modulation of AhR is required for oligodendrocyte precursor cell development as well as for brain development. At the same time, based on morphological descriptors, the toxicity of the endogenous potent AhR agonist 6-formylindolo[3,2-b]carbazole (FICZ) was only marginal in controls and enhanced in the *cyp1a* knockdown zebrafish larvae [77]. Majewski et al. [78] investigated the acute toxicity of the KYN metabolites on zebrafish embryos and larvae. Among the tested compounds, KYN seemed to be safe because the authors were not able to determine its toxic concentration, and the only visible difference compared to controls was an increased heartbeat. The involvement of the AhR in this phenomenon has not been studied. In our experimental setup, the KYN concentration used neither induced morphological toxicity nor affected muscle function and performance.

In this study, we exposed zebrafish embryos and larvae to KYN at different periods and analyzed their behavior at 5 dpf. Here, the hypoactivity at 5 dpf was observed only in those groups that were incubated in KYN between 1 and 72 hpf and between 49 and 72 hpf. About 6 hpf, neurodevelopment in zebrafish embryos begins during the gastrula period when the neural plate is already formed. At this time, there also occur cells that will later form neurons in the hindbrain, the notochord, and somite-derived muscles. At 24 hpf, the zebrafish brain is complete and divided into the forebrain, the midbrain, and the hindbrain, with the initial neurons interconnected by axons [51,79,80]. Thus, this period, i.e., from cleavage to segmentation (1–24 hpf), seems to be critically important for proper zebrafish neurodevelopment. Recently, Liu et al. [81] showed that the spontaneous locomotor activity of four dpf larvae exposed to bisphenol A between 1–24 hpf was decreased compared to control larvae. This was associated with reduced mRNA expression of guanine deaminase (cypin) within this time because the injection of cypin mRNA abolished these locomotor activity disturbances. Interestingly, four dpf larvae exposed to bisphenol A in later periods (i.e., 24–48 hpf and 48–72 hpf) did not exhibit locomotor activity disturbances.

Taking all the above into account, it is very challenging to explain behavioral changes in the larvae exposed to KYN during the period when the neuronal tube is closed and the period of intensive formation of the central nervous system is initiated. Whereas KYN exposure from 1 to 24 hpf and from 25 to 48 hpf did not affect zebrafish behavior, we cannot rule out that these changes indeed occurred, but the tests used by us were not sensitive enough to detect these subtle disturbances. Since zebrafish is an excellent model for genetic manipulation, it gives us the opportunity to determine, in future work, which AhR-mediated, extremely complex mechanisms are engaged in the KYN-related behavioral disturbances in larval zebrafish.

Considering that in our study the exposure to KYN was conducted in aqueous solution at 28.5 °C temperature and lasted for 24 h, it is important to evidence whether KYN was not degraded under these conditions. Therefore, we measured the KYN content in the incubation medium after each 24-hour incubation period. We found that in the medium without the presence of larvae, the KYN content did not change statistically significantly, which is in line with the data obtained under comparable conditions by Schwieler et al. [82] and Hu et al. [83]. Moreover, our study revealed that the content of KYN in the zebrafish medium incubated with larvae from 96 to 119 hpf did not decrease significantly compared to the medium without larvae (control), which indicates that the larvae do not metabolize KYN meaningfully and allows us to conclude that the observed changes are due to KYN.

However, during the search for KYNA in the post-incubation medium, the presence of unidentified peaks was also noted. The presence of a KYNA-like substance was determined by retention time and after adding the authentic KYNA to the medium sample. In both cases, the retention time was identical to that of the KYNA standard, which seems to confirm that larvae synthesize KYNA from KYN. The estimated production of KYNA is very low, and although KYNA is a more potent AhR agonist than KYN [84], it seems unlikely that this metabolite is the cause of behavioral disturbances observed in our study.

Disregarding the low concentration of KYNA in the medium, its action by another receptor mechanism also seems unlikely, although two GPR35 paralogs have been shown to exist in zebrafish, *gpr35a* with 25.6% and *gpr35b* with 24% identity to the human GPR35 protein sequence [85]. The authors showed that *gpr35a* was even expressed in the gut bulb and the rest of the body at 120 hpf, although they did not specify whether *gpr35a* was expressed in the brain and in what structures. In the case of *grp35b*, expression was restricted to the intestinal bulb only. Both zaprinast and lysophosphatidic acid, GPR35 agonists, when administered to zebrafish larvae, caused inflammation and are therefore considered active ligands in this species [86]. Unfortunately, KYNA was not tested in this experimental setup. Since interspecies differences in the activity of GPR35 agonists are known, it is not certain whether KYNA would be a pharmacologically active ligand in zebrafish larvae.

In addition to the KYNA-like substance, the chromatograms obtained under optimal conditions for KYNA detection showed at least three remarkable peaks that appear only in the presence of larvae, suggesting that these are other KYN metabolites formed by larvae. Identification of these substances requires further research. However, it cannot be ruled out that those KYN metabolites may be highly biologically active. Seok et al. [87] demonstrated that incubation of KYN in a solution in conditions similar to ours can increase its effect on AhR 100–1000 times. In addition, purified trace active derivatives of KYN have been described, and two new condensation products have been identified, named trace extended aromatic condensation products (TEACOP), active at low picomolar levels [87].

Taken together, the presence of substances other than KYN in the medium inhabited by larvae indicates that KYN is absorbed from the medium, metabolized in the larvae, and the metabolites are excreted into the medium. These findings imply that the zebrafish medium system forms an active biological compartment that enables the monitoring of drug metabolism in zebrafish.

Since the incubation of zebrafish embryos and larvae early in life with KYN indicated some behavioral disturbances, we decided to investigate how the administration of KYN early in rodent pregnancy would affect the offspring. Our study revealed that KYN supplementation to dams on ED 1–7 resulted in slight alterations in the offspring’s neurodevelopment. No effect of KYN supplementation on either the pregnancy course or its outcome was observed. All pregnancies were full-term, and the number of offspring and birth weight were comparable with the corresponding control group.

However, the biochemical analysis of the blood plasma of mothers and young offspring after breastfeeding and adult offspring indicated significant differences. The intragastric administration of KYN to pregnant dams on ED 1–7 resulted in increased levels of tryptophan, KYN, and KYNA in blood plasma of mothers at 21 days postpartum. The level of KYN in young offspring was elevated, while in adults, it was unchanged. KYNA content was decreased in young male offspring only. The reason for such alterations is unknown. It can be hypothesized that excess KYN caused a change in the functioning of mechanisms regulating the activity of the tryptophan metabolism pathway along the kynurenine pathway, not unlike the serotonin pathway, which, however, was not investigated in this study. Regardless of the underlying molecular mechanism, the signaled changes suggest that KYN may affect the development of the organism not only directly through AhR receptors but that these changes may also be a secondary consequence of profound and persistent alteration of tryptophan metabolism.

Another difference that was noticeable during the development of animals was that the offspring of the dams supplemented with KYN gained less body weight, which concerned primarily females. Although numerous studies have been conducted on KYN supplementation in pregnant dams, other authors have not reported such differences in offspring weight gain [25,26,27,28,29,30,31,88,89]. This effect does not appear to have contributed to behavior changes, as the body weight reduction on the day of behavior testing was 8% in females and was non-significant in males.

Thus, when analyzing the effect of KYN administration on ED 1–7 on the behavior of adult offspring, it was found that the spontaneous locomotor activity of adult offspring male rats was unchanged, while the vertical activity of adult female offspring rats was reduced. To further investigate the susceptibility of adult offspring rats, two stimulants, i.e., amphetamine and dizocilpine were administered. It was established that KYN administration on ED 1–7 reduced the susceptibility of adult offspring of male and female rats to the action of dizocylpine and had little effect on the action of amphetamine. Furthermore, adult male offspring rats attempted more entries into the previous escape location in reversal learning during the Barnes maze task while other parameters remained unchanged. There was no effect of KYN administration on ED 1–7 on anxiety-like behavior and associative learning in adult offspring rats. The reason for this late manifestation of KYN administration in early pregnancy is unknown. Changes in animal behavior are unexpected as the rat brain development occurs later, on ED 10.5–11 [39,90].

Our behavioral findings are consistent with the data presented in the series of publications in which rats were exposed to parenterally infused KYN, either pre- or postnatally (see the review) [91]. In our previous study, KYN administration during the breastfeeding period changed the stimulated locomotor activity and impaired cognitive flexibility and contextual fear conditioning in male offspring rats [32]. In that study, changes in the stimulated locomotor activity were reported in both sexes, more pronounced after dizocylpine. Dizocylpine, a non-competitive NMDA antagonist used in our study, produced hyperlocomotor effects considered to be an experimental model of psychosis [92], while deficits in memory processes have been suggested to be highly useful models of cognitive impairment in schizophrenia [93]. Blockade of NMDA receptors by dizocylpine leads to disruption of glutamatergic neurotransmission, and dopaminergic neurotransmission is not required for the effects of NMDA antagonists [94]; however, in conditions where the kynurenine pathway has been interfered with, it cannot be ruled out.

Furthermore, body weight gain and spontaneous vertical activity were reduced in female adult offspring. These parameters may indicate depressive-related behavior as exposure to chronic unpredictable mild stress increased depressive-related behavior and decreased the serum brain-derived neurotrophic factor in female rats but not in male rats [95]. In addition, some depressive behaviors may occur only in females as early-life stress-induced depressive-like behavior in female mice but not in male mice [96]. The higher rate of depression in females vs. males may be related to alterations in the kynurenine pathway metabolism that increase vulnerability to depression in females [97]. It has been indicated that estrogen exerts an inhibitory effect on KAT enzymes, leading to a decrease in KYNA production [98]. In contrast to depression, the prevalence of schizophrenia in males is ∼2.5 times greater than in females [99]. An elevated level of KYNA in the brain has been hypothesized to be a pathophysiological mechanism underlying schizophrenia [100,101] and estrogen-related decreases in KYNA may be protective, possibly partly explaining why females are less likely to develop schizophrenia-related disorders [102].

To date, males have attracted attention in terms of their use in animal models due to concerns about confounding factors of the hormonal cycle changes in females. However, studies have shown that the variability observed in female mice throughout the hormonal cycle is not greater than that in males [103]. Moreover, data obtained in males may not be generalizable to psychopathology and drug treatment efficacy in females [104,105]. Therefore, further research is needed to elucidate this phenomenon.

In this study, we found that exposure to KYN in both zebrafish and rats resulted in late changes in animal behavior, but the molecular mechanism of KYN action has not been investigated. In our research, we increased the amount of KYN in the zebrafish larval environment or administered it to pregnant rats. Since KYN is readily transported through the intestinal barrier and crosses the placenta and the fetal blood-brain, it can be concluded that the central nervous system disturbances may result from an excess of KYN penetrating the placental barrier. The AhR receptor, with KYN being its ligand, is present in early developmental tissues, including the brain [18]. Indeed, it has been indicated that at E13.5, AhR mRNA is expressed at high levels in rodents in the primitive pituitary, the palatal shelf, the nasal septal cartilage, the dorsal surface of the tongue, the developing thymus, the lung parenchyma, the liver, the mucosa of the developing gut, the kidney, the urogenital sinus, and the tip of the genital tubercle [106].

Furthermore, it should be recalled that, in addition to its direct action on the AhR receptor, KYN is a precursor of many metabolites that have some biological effects. One of them is KYNA, which is an AhR agonist, as well as a GPR35 agonist and glutamatergic receptor antagonist [16,18,107]. Elevated brain levels of KYNA appear to be associated with psychotic symptoms and cognitive impairments [8]. Another biologically active metabolite of KYN is 3-hydroxykynurenine, which crosses blood–brain barrier and placenta, is redox-active, and may regulate and maintain the oxidative status of tissues in order to prevent cell damage by reactive oxygen species [108]. However, under pathological conditions, 3-hydroxykynurenine generates highly reactive free radicals and, thereby, potentiates the neurotoxic effects of quinolinic acid [109]. Quinolinic acid acts as a neurotoxin, gliotoxin, proinflammatory mediator, and pro-oxidant molecule and can alter the integrity and cohesion of the blood–brain barrier [110]. In addition, KYNA and quinolinic acid, whether derived from the mother or the placenta, may be able to enter the fetal brain relatively freely in the prenatal period. Xanthurenic acids, like KYN and KYNA, are AhR ligands and possess potential antioxidant activity by maintaining redox homeodynamics [111]. Xanthurenic acid may modulate glutamatergic neurotransmission by inhibiting the vesicular glutamate transporter and/or activating group II metabotropic glutamate receptors. These properties of xanthurenic acid may be of special relevance in the pathophysiology of psychiatric disorders [112].

Therefore, it is difficult to conclude whether KYN has a direct or indirect effect attributed to its metabolites on the developing organism. In this context, the possibility of conducting neurotoxicological studies on zebrafish, which we have demonstrated in this paper, is a great advantage because the larval zebrafish model is very convenient for selective gene knockout or silencing, which allows for precise determination of the role of individual receptors in the early development of the brain.

In our rat study, KYN was administered orally. It is not known whether there is a risk of overdosing KYN with food, as there is little data on the food content of this ingredient [91]. A real problem may be the oversupply of tryptophan in foods and supplements. It is believed that the amount of tryptophan consumed in the USA is about three times higher than the daily requirement for this amino acid [113]. Since approximately 95% of absorbed tryptophan is converted along the kynurenine pathway, one can imagine that the amount of KYN and its metabolites significantly exceeds physiological values. This can also affect pregnant women, and this problem seems to be overlooked. In a study conducted by Castrogiovanni et al. [114] and Musumeci et al. [115], pregnant rats had free access to high-tryptophan chow from the first day of pregnancy. Their offspring showed morphological changes in muscle fibers and reduced expression of insulin-like growth factor I (IGF-I) in the liver and muscle tissue. The muscle tissue was less developed and defined, which affected body mass, the average weight of pups, and their survival rate.

The activation of the kynurenine pathway also occurs under inflammation and stress conditions, as both these factors activate the enzymes that convert tryptophan to KYN [4]. It is generally recognized that stressful events during pregnancy can significantly affect the development of the central nervous system and the subsequent functioning of the offspring’s brain. It has been indicated that maternal stress increases the incidence of schizophrenia [116], attention-deficit hyperactivity disorder, and memory deficits [117,118,119] in individuals exposed in utero to stressful episodes [120]. Interestingly, the long-term effects of exposure to intra-utero stress may vary by gender. Men exposed to prenatal stress show a higher incidence of schizophrenia [121] and attention-deficit hyperactivity disorder [122,123], while women exposed to prenatal stress have been reported to have an increased susceptibility to depression, and there is a correlation between depression and lower birth weight [124,125]. Stress conditions impair immunological functions [126,127] and induce the expression of proinflammatory cytokines, which stimulate the kynurenine pathway by increasing the expression of IDO [128,129]. Under these circumstances, KYN is more likely to be metabolized to several neuroactive compounds that are directly linked to the pathophysiology of anxiety, depression, and schizophrenia [130,131,132].

The limitation of the presented data is the lack of comprehensive knowledge of the hormonal status in subjected female rats. Vaginal cytology was not collected on the test days to minimize handling stress. However, to match the estrous cycle stages within subjects, efforts were made to conduct behavioral tests during the 4-day cycle of rats [133]. Since there are significant changes in biochemical results, gene expression, and enzyme activity should be investigated in a similar experimental setup. The lack of immunohistological assessment of the receptors targeted by KYN and other tryptophan metabolites should be considered a limitation of our study, thus making further research imperative.

In conclusion, our study has revealed that exposure to KYN affects the early stages of development, and excessive KYN supplementation should be avoided even at this early stage of development. Incubation in the medium with KYN changed zebrafish behavior, especially when zebrafish embryos or larvae were incubated with KYN from 1 to 72 hpf and from 49 to 72 hpf. Zebrafish is able to metabolize KYN, and together with the medium, it forms an active biological system. Moreover, KYN administered early in pregnancy to dam rats (ED 1–7) induced slight differences in the neurobehavioral development of the adult offspring, with female offspring being more sensitive to KYN. Further research is required to understand the mechanism of these changes. Our finding proves that the larval zebrafish model can be useful for studying disturbances of early brain development processes and their late behavioral consequences. Nonetheless, the appropriate age of the larvae and more sensitive tests should be taken into account. The zebrafish medium system may be useful in monitoring drug metabolism in zebrafish.

## Figures and Tables

**Table 1:** Effect of KYN administration during ED 1–7 on the level of tryptophan, KYN, and KYNA in animal plasma.

Compound	Mother	Young Offspring		Adult Offspring	
		Male	Female	Male	Female
Tryptophan	↑	↓	=	↓	↓
KYN	↑	↑	↑	=	=
KYNA	↑	↓	=	=	=

= no effect. ↓ decrease. ↑ increase; see Tables S1–S3 for details. KYN—kynurenine, KYNA—kynurenic acid.

**Table 2:** Effect of KYN administration during ED 1–7 on locomotor activity of adult male rats.

		Spontaneous	Amphetamine	Dizocylpine 0.15	Dizocylpine 0.3
1	Horizontal activity	=	=	=	↓
2	Total distance (cm)	=	=	=	↓
3	No. of movements	=	=	=	=
4	Movement time (s)	=	=	=	↓
5	Rest time (s)	=	↑	=	↑
6	Vertical activity	=	=	=	=
7	No. of vertical movements	=	=	=	=
8	Vertical time (s)	=	↓	=	=
9	Stereotypy counts	=	=	=	↓
10	No. of stereotypy	=	↓	=	=
11	Stereotypy time (s)	=	=	=	↓
12	Margin distance (cm)	=	=	=	=
13	Margin time (s)	=	=	=	=
14	Center distance (cm)	=	=	=	=
15	Center time (s)	=	=	=	=
16	Horizontal activity + Vertical activity	=	=	=	↓
17	Horizontal activity/Vertical activity	=	=	=	=
18	No of movements + No of vertical movements	=	=	=	=
19	No of movements/No of vertical movements	=	=	=	=
20	Movement time + Vertical time	=	=	=	↓
21	Movement time/Vertical time	=	=	=	=
22	Horizontal activity + Vertical activity/Stereotypy counts	=	↑	=	↑
23	No of movements + No of vertical movements/No of stereotypy	=	=	=	=
24	Movement time + Vertical time/stereotypy time	=	↑	=	=
25	Marigin distance/Center distance	=	=	=	=
26	Marigin time/Center time	=	=	=	=

= no effect. ↓ decrease. ↑ increase.

**Table 3:** Effect of KYN administration during ED 1–7 on locomotor activity of adult female rats.

		Spontaneous	Amphetamine	Dizocylpine 0.075	Dizocylpine 0.15
1	Horizontal activity	=	=	=	=
2	Total distance (cm)	=	=	=	=
3	No. of movements	=	=	=	=
4	Movement time (s)	=	=	=	=
5	Rest time (s)	=	=	=	=
6	Vertical activity	↓	=	=	↓
7	No. of vertical movements	↓	=	=	↓
8	Vertical time (s)	↓	=	=	↓
9	Stereotypy counts	=	=	↑	=
10	No. of stereotypy	=	=	=	=
11	Stereotypy time (s)	=	=	↑	=
12	Margin distance (cm)	=	=	=	=
13	Margin time (s)	↑	=	=	↑
14	Center distance (cm)	=	=	=	↓
15	Center time (s)	=	=	=	↓
16	Horizontal activity + Vertical activity	=	=	=	=
17	Horizontal activity/Vertical activity	↑	=	=	=
18	No of movements + No of vertical movements	=	=	=	=
19	No of movements/No of vertical movements	=	=	=	=
20	Movement time + Vertical time	↓	=	=	=
21	Movement time/Vertical time	↑	=	=	=
22	Horizontal activity + Vertical activity/Stereotypy counts	=	↑	↓	=
23	No of movements + No of vertical movements/No of stereotypy	↓	=	=	=
24	Movement time + Vertical time/Stereotypy time	↓	=	=	=
25	Marigin distance/Center distance	=	=	=	↑
26	Marigin time/Center time	=	=	=	=

= no effect. ↓ decrease. ↑ increase.

## Data Availability

Data sharing is not applicable to this article.

## Supplementary Materials

The following supporting information can be downloaded at: https://www.mdpi.com/article/10.3390/cells12182224/s1, Figure S1: Locomotor activity under the light-dark transition assay of 5-day-old larval zebrafish exposed to KYN from 1 to 72 (A–D) and from 49 to 72 (E–H) hpf; Figure S2: Evidence for the formation of KYN metabolites by zebrafish larvae; Figure S3: Identification of KYNA-like substance in zebrafish incubation medium by comparison of retention time of peaks in samples with authentic KYNA; Figure S4: Identification of KYNA-like substance in zebrafish incubation medium by addition of an internal standard to the original sample; Figure S5: Effect of KYN administration during ED 1–7 on the body mass gain of male (A) and female (B) rats; Figure S6: Effect of KYN administration during ED 1–7 on the spontaneous and stimulated locomotor activity of adult male rats; Figure S7: Effect of KYN administration during ED 1–7 on the spontaneous and stimulated locomotor activity of adult female rats; Figure S8: Effect of KYN administration during ED 1–7 on the anxiety-like behavior of adult rats, estimated in the elevated plus-maze test; Figure S9: Effect of KYN administration during ED 1–7 on the reversal learning of adult rats; Figure S10: Effect of KYN administration during ED 1–7 on the associative learning of adult rats; Table S1: Effect of KYN administration during ED 1–7 on the level of tryptophan, KYN and KYNA in mother’s plasma; Table S2: Effect of KYN administration during ED 1–7 on the level of tryptophan, KYN and KYNA in male offspring’s plasma; Table S3: Effect of KYN administration during ED 1–7 on the level of tryptophan, KYN and KYNA in female offspring’s plasma; Table S4: Effect of KYN administration during ED 1–7 on anxiety-like behavior and memory processes of adult male rats; Table S5: Effect of KYN administration during ED 1–7 on anxiety-like behavior and memory processes of adult female rats.

## References

[1] Hu D., Liu J., Yu W., Li C., Huang L., Mao W., Lu Z. (2023). Tryptophan intake, not always the more the better. Front. Nutr..

[2] King N.J., Thomas S.R. (2007). Molecules in focus: Indoleamine 2,3-dioxygenase. Int. J. Biochem. Cell Biol..

[3] Capece L., Lewis-Ballester A., Marti M.A., Estrin D.A., Yeh S.R. (2011). Molecular basis for the substrate stereoselectivity in tryptophan dioxygenase. Biochemistry.

[4] Cervenka I., Agudelo L.Z., Ruas J.L. (2017). Kynurenines: Tryptophan’s metabolites in exercise, inflammation, and mental health. Science.

[5] Leklem J.E. (1971). Quantitative aspects of tryptophan metabolism in humans and other species: A review. Am. J. Clin. Nutr..

[6] Badawy A.A. (2014). The tryptophan utilization concept in pregnancy. Obstet. Gynecol. Sci..

[7] Teshigawara T., Mouri A., Kubo H., Nakamura Y., Shiino T., Okada T., Morikawa M., Nabeshima T., Ozaki N., Yamamoto Y. (2019). Changes in tryptophan metabolism during pregnancy and postpartum periods: Potential involvement in postpartum depressive symptoms. J. Affect. Disord..

[8] Erhardt S., Schwieler L., Imbeault S., Engberg G. (2017). The kynurenine pathway in schizophrenia and bipolar disorder. Neuropharmacology.

[9] Notarangelo F.M., Pocivavsek A. (2017). Elevated kynurenine pathway metabolism during neurodevelopment: Implications for brain and behavior. Neuropharmacology.

[10] Schwarcz R., Bruno J.P., Muchowski P.J., Wu H.Q. (2012). Kynurenines in the mammalian brain: When physiology meets pathology. Nat. Rev. Neurosci..

[11] Stone T.W. (1993). Neuropharmacology of quinolinic and kynurenic acids. Pharmacol. Rev..

[12] Stone T.W. (2007). Kynurenic acid blocks nicotinic synaptic transmission to hippocampal interneurons in young rats. Eur. J. Neurosci..

[13] Hilmas C., Pereira E.F., Alkondon M., Rassoulpour A., Schwarcz R., Albuquerque E.X. (2001). The brain metabolite kynurenic acid inhibits alpha7 nicotinic receptor activity and increases non-alpha7 nicotinic receptor expression: Physiopathological implications. J. Neurosci..

[14] Stone T.W. (2020). Does kynurenic acid act on nicotinic receptors? An assessment of the evidence. J. Neurochem..

[15] Oxenkrug G., van der Hart M., Roeser J., Summergrad P. (2016). Anthranilic acid: A potential biomarker and treatment target for schizophrenia. Ann. Psychiatry Ment. Health.

[16] Wang J., Simonavicius N., Wu X., Swaminath G., Reagan J., Tian H., Ling L. (2006). Kynurenic acid as a ligand for orphan G protein-coupled receptor GPR35. J. Biol. Chem..

[17] Shore D.M., Reggio P.H. (2015). The therapeutic potential of orphan GPCRs, GPR35 and GPR55. Front. Pharmacol..

[18] DiNatale B.C., Murray I.A., Schroeder J.C., Flaveny C.A., Lahoti T.S., Laurenzana E.M., Omiecinski C.J., Perdew G.H. (2010). Kynurenic acid is a potent endogenous aryl hydrocarbon receptor ligand that synergistically induces interleukin-6 in the presence of inflammatory signaling. Toxicol. Sci..

[19] Yamamoto J., Ihara K., Nakayama H., Hikino S., Satoh K., Kubo N., Iida T., Fujii Y., Hara T. (2004). Characteristic expression of aryl hydrocarbon receptor repressor gene in human tissues: Organ-specific distribution and variable induction patterns in mononuclear cells. Life Sci..

[20] Murray I.A., Patterson A.D., Perdew G.H. (2014). Aryl hydrocarbon receptor ligands in cancer: Friend and foe. Nat. Rev. Cancer.

[21] Stone T., Williams R.O. (2023). Interactions of IDO and the kynurenine pathway with cell transduction Systems and metabolism at the inflammation–cancer interface. Cancers.

[22] Turska M., Paluszkiewicz P., Turski W.A., Parada-Turska J. (2022). A review of the health benefits of food enriched with kynurenic acid. Nutrients.

[23] Solvay M., Holfelder P., Klaessens S., Pilotte L., Stroobant V., Lamy J., Naulaerts S., Spillier Q., Frédérick R., De Plaen E. (2023). Tryptophan depletion sensitizes the AHR pathway by increasing AHR expression and GCN2/LAT1-mediated kynurenine uptake, and potentiates induction of regulatory T lymphocytes. J. Immunother. Cancer.

[24] Goeden N., Notarangelo F.M., Pocivavsek A., Beggiato S., Bonnin A., Schwarcz R. (2017). Prenatal dynamics of kynurenine pathway metabolism in mice: Focus on kynurenic acid. Dev. Neurosci..

[25] Pershing M.L., Bortz D.M., Pocivavsek A., Fredericks P.J., Jørgensen C.V., Vunck S.A., Leuner B., Schwarcz R., Bruno J.P. (2015). Elevated levels of kynurenic acid during gestation produce neurochemical, morphological, and cognitive deficits in adulthood: Implications for schizophrenia. Neuropharmacology.

[26] Pocivavsek A., Wu H.Q., Elmer G.I., Bruno J.P., Schwarcz R. (2012). Pre- and postnatal exposure to kynurenine causes cognitive deficits in adulthood. Eur. J. Neurosci..

[27] Pocivavsek A., Thomas M.A., Elmer G.I., Bruno J.P., Schwarcz R. (2014). Continuous kynurenine administration during the prenatal period, but not during adolescence, causes learning and memory deficits in adult rats. Psychopharmacology.

[28] Pocivavsek A., Elmer G.I., Schwarcz R. (2019). Inhibition of kynurenine aminotransferase II attenuates hippocampus-dependent memory deficit in adult rats treated prenatally with kynurenine. Hippocampus.

[29] Alexander K.S., Pocivavsek A., Wu H.Q., Pershing M.L., Schwarcz R., Bruno J.P. (2013). Early developmental elevations of brain kynurenic acid impair cognitive flexibility in adults: Reversal with galantamine. Neuroscience.

[30] Pershing M.L., Phenis D., Valentini V., Pocivavsek A., Lindquist D.H., Schwarcz R., Bruno J.P. (2016). Prenatal kynurenine exposure in rats: Age-dependent changes in NMDA receptor expression and conditioned fear responding. Psychopharmacology.

[31] Milosavljevic S., Smith A.K., Wright C.J., Valafar H., Pocivavsek A. (2023). Kynurenine aminotransferase II inhibition promotes sleep and rescues impairments induced by neurodevelopmental insult. Transl. Psychiatry.

[32] Marszalek-Grabska M., Stachniuk A., Iwaniak P., Gawel K., Sumara A., Kocki T., Fornal E., Milart P., Paluszkiewicz P., Turski W. (2022). Unexpected content of kynurenine in mother’s milk and infant formulas. Sci. Rep..

[33] Forrest C.M., Khalil O.S., Pisar M., Darlington L.G., Stone T.W. (2013). Prenatal inhibition of the tryptophan-kynurenine pathway alters synaptic plasticity and protein expression in the rat hippocampus. Brain Res..

[34] Pisar M., Forrest C.M., Khalil O.S., McNair K., Vincenten M.C., Qasem S., Darlington L.G., Stone T.W. (2014). Modified neocortical and cerebellar protein expression and morphology in adult rats following prenatal inhibition of the kynurenine pathway. Brain Res..

[35] Khalil O.S., Pisar M., Forrest C.M., Vincenten M.C., Darlington L.G., Stone T.W. (2014). Prenatal inhibition of the kynurenine pathway leads to structural changes in the hippocampus of adult rat offspring. Eur. J. Neurosci..

[36] Erhardt S., Pocivavsek A., Repici M., Liu X.C., Imbeault S., Maddison D.C., Thomas M.A.R., Smalley J.L., Larsson M.K., Muchowski P.J. (2017). Adaptive and behavioral changes in kynurenine 3-monooxygenase knockout mice: Relevance to psychotic disorders. Biol. Psychiatry.

[37] Mu J., Zhou Z., Sang Q., Wang L. (2022). The physiological and pathological mechanisms of early embryonic development. Fundam. Res..

[38] DeSesso J.M., Scialli A.R., Holson J.F. (1999). Apparent lability of neural tube closure in laboratory animals and humans. Am. J. Med. Genet..

[39] Rice D., Barone S. (2000). Critical periods of vulnerability for the developing nervous system: Evidence from humans and animal models. Environ. Health Perspect..

[40] Clarke J. (2009). Role of polarized cell divisions in zebrafish neural tube formation. Curr. Opin. Neurobiol..

[41] Pla P., Monsoro-Burq A.H. (2018). The neural border: Induction, specification and maturation of the territory that generates neural crest cells. Dev. Biol..

[42] Gawel K., Langlois M., Martins T., van der Ent W., Tiraboschi E., Jacmin M., Crawford A.D., Esguerra C.V. (2020). Seizing the moment: Zebrafish epilepsy models. Neurosci. Biobehav. Rev..

[43] Howe K., Clark M.D., Torroja C.F., Torrance J., Berthelot C., Muffato M., Collins J.E., Humphray S., McLaren K., Matthews L. (2013). The zebrafish reference genome sequence and its relationship to the human genome. Nature.

[44] Gawel K., Banono N.S., Michalak A., Esguerra C.V. (2019). A critical review of zebrafish schizophrenia models: Time for validation?. Neurosci. Biobehav. Rev..

[45] Jeong J.Y., Kwon H.B., Ahn J.C., Kang D., Kwon S.H., Park J.A., Kim K.W. (2008). Functional and developmental analysis of the blood-brain barrier in zebrafish. Brain Res. Bull..

[46] Fleming A., Diekmann H., Goldsmith P. (2013). Functional characterisation of the maturation of the blood-brain barrier in larval zebrafish. PLoS ONE.

[47] Zhao J., Gao P., Zhu D. (2010). Optimization of Zn^2+^-containing mobile phase for simultaneous determination of kynurenine, kynurenic acid and tryptophan in human plasma by high performance liquid chromatography. J. Chromatogr. B Analyt. Technol. Biomed. Life Sci..

[48] Gawel K., Turski W.A., van der Ent W., Mathai B.J., Kirstein-Smardzewska K.J., Simonsen A., Esguerra C.V. (2020). Phenotypic characterization of larval zebrafish (*Danio rerio*) with partial knockdown of the cacna1a gene. Mol. Neurobiol..

[49] Nakonieczna S., Grabarska A., Gawel K., Wróblewska-Łuczka P., Czerwonka A., Stepulak A., Kukula-Koch W. (2022). Isoquinoline alkaloids from coptis chinensis Franch: Focus on coptisine as a potential therapeutic candidate against gastric cancer cells. Int. J. Mol. Sci..

[50] Gawel K., Kukula-Koch W., Banono N.S., Nieoczym D., Targowska-Duda K.M., Czernicka L., Parada-Turska J., Esguerra C.V. (2021). 6-Gingerol, a major constituent of zingiber officinale rhizoma, exerts anticonvulsant activity in the pentylenetetrazole-induced seizure model in larval zebrafish. Int. J. Mol. Sci..

[51] Kimmel C.B., Ballard W.W., Kimmel S.R., Ullmann B., Schilling T.F. (1995). Stages of embryonic development of the zebrafish. Dev. Dyn..

[52] Préau L., Fini J.B., Morvan-Dubois G., Demeneix B. (2015). Thyroid hormone signaling during early neurogenesis and its significance as a vulnerable window for endocrine disruption. Biochim. Biophys. Acta.

[53] Banono N.S., Gawel K., De Witte L., Esguerra C.V. (2021). Zebrafish larvae carrying a splice variant mutation in cacna1d: A new model for schizophrenia-like behaviours?. Mol. Neurobiol..

[54] Faria M., Bellot M., Soto O., Prats E., Montemurro N., Manjarrés D., Gómez-Canela C., Raldúa D. (2022). Developmental exposure to sertraline impaired zebrafish behavioral and neurochemical profiles. Front. Physiol..

[55] Burton C.E., Zhou Y., Bai Q., Burton E.A. (2017). Spectral properties of the zebrafish visual motor response. Neurosci. Lett..

[56] Klüver N., König M., Ortmann J., Massei R., Paschke A., Kühne R., Scholz S. (2015). Fish embryo toxicity test: Identification of compounds with weak toxicity and analysis of behavioral effects to improve prediction of acute toxicity for neurotoxic compounds. Environ. Sci. Technol..

[57] Irons T.D., MacPhail R.C., Hunter D.L., Padilla S. (2010). Acute neuroactive drug exposures alter locomotor activity in larval zebrafish. Neurotoxicol. Teratol..

[58] Souders C.L., Davis R.H., Qing H., Liang X., Febo M., Martyniuk C.J. (2019). The psychoactive cathinone derivative pyrovalerone alters locomotor activity and decreases dopamine receptor expression in zebrafish (*Danio rerio*). Brain Behav..

[59] Peng X., Lin J., Zhu Y., Liu X., Zhang Y., Ji Y., Yang X., Zhang Y., Guo N., Li Q. (2016). Anxiety-related behavioral responses of pentylenetetrazole-treated zebrafish larvae to light-dark transitions. Pharmacol. Biochem. Behav..

[60] Maeda H., Hasumi A., Yoshida K.I. (2021). Caffeine-induced bradycardia, death, and anxiety-like behavior in zebrafish larvae. Forensic Toxicol..

[61] Yang X., Lin J., Peng X., Zhang Q., Zhang Y., Guo N., Zhou S., Li Q. (2017). Effects of picrotoxin on zebrafish larvae behaviors: A comparison study with PTZ. Epilepsy Behav..

[62] Beischlag T.V., Luis Morales J., Hollingshead B.D., Perdew G.H. (2008). The aryl hydrocarbon receptor complex and the control of gene expression. Crit. Rev. Eukaryot. Gene Expr..

[63] Tanguay R.L., Abnet C.C., Heideman W., Peterson R.E. (1999). Cloning and characterization of the zebrafish (*Danio rerio*) aryl hydrocarbon receptor. Biochim. Biophys. Acta (BBA)-Gene Struct. Expr..

[64] Andreasen E.A., Hahn M.E., Heideman W., Peterson R.E., Tanguay R.L. (2002). The zebrafish (*Danio rerio*) aryl hydrocarbon receptor type 1 is a novel vertebrate receptor. Mol. Pharmacol..

[65] Hahn M.E., Karchner S.I., Merson R.R. (2017). Diversity as opportunity: Insights from 600 million years of AHR evolution. Curr. Opin. Toxicol..

[66] Shankar P., Dasgupta S., Hahn M.E., Tanguay R.L. (2020). A review of the functional roles of the zebrafish aryl hydrocarbon receptors. Toxicol. Sci..

[67] Sugden W.W., Leonardo-Mendonça R.C., Acuña-Castroviejo D., Siekmann A.F. (2017). Genetic dissection of endothelial transcriptional activity of zebrafish aryl hydrocarbon receptors (AHRs). PLoS ONE.

[68] Garcia G.R., Bugel S.M., Truong L., Spagnoli S., Tanguay R.L. (2018). AHR2 required for normal behavioral responses and proper development of the skeletal and reproductive systems in zebrafish. PLoS ONE.

[69] Mathew L.K., Andreasen E.A., Tanguay R.L. (2006). Aryl hydrocarbon receptor activation inhibits regenerative growth. Mol. Pharmacol..

[70] Kubota A., Goldstone J.V., Lemaire B., Takata M., Woodin B.R., Stegeman J.J. (2015). Role of pregnane X receptor and aryl hydrocarbon receptor in transcriptional regulation of pxr, CYP2, and CYP3 genes in developing zebrafish. Toxicol. Sci..

[71] Prasch A.L., Tanguay R.L., Mehta V., Heideman W., Peterson R.E. (2006). Identification of zebrafish ARNT1 homologs: 2,3,7,8-tetrachlorodibenzo-p-dioxin toxicity in the developing zebrafish requires ARNT1. Mol. Pharmacol..

[72] Antkiewicz D.S., Peterson R.E., Heideman W. (2006). Blocking expression of AHR2 and ARNT1 in zebrafish larvae protects against cardiac toxicity of 2,3,7,8-tetrachlorodibenzo-p-dioxin. Toxicol. Sci..

[73] Incardona J.P., Day H.L., Collier T.K., Scholz N.L. (2006). Developmental toxicity of 4-ring polycyclic aromatic hydrocarbons in zebrafish is differentially dependent on AH receptor isoforms and hepatic cytochrome P4501A metabolism. Toxicol. Appl. Pharmacol..

[74] Mimura J., Fujii-Kuriyama Y. (2003). Functional role of AhR in the expression of toxic effects by TCDD. Biochim. Biophys. Acta (BBA)-Gen. Subj..

[75] Carney S.A., Prasch A.L., Heideman W., Peterson R.E. (2006). Understanding dioxin developmental toxicity using the zebrafish model. Birth Defects Res. Part A Clin. Mol. Teratol..

[76] Martin N.R., Patel R., Kossack M.E., Tian L., Camarillo M.A., Cintrón-Rivera L.G., Gawdzik J.C., Yue M.S., Nwagugo F.O., Elemans L.M.H. (2022). Proper modulation of AHR signaling is necessary for establishing neural connectivity and oligodendrocyte precursor cell development in the embryonic zebrafish brain. Front. Mol. Neurosci..

[77] Wincent E., Kubota A., Timme-Laragy A., Jönsson M.E., Hahn M.E., Stegeman J.J. (2016). Biological effects of 6-formylindolo[3,2-b]carbazole (FICZ) in vivo are enhanced by loss of CYP1A function in an Ahr2-dependent manner. Biochem. Pharmacol..

[78] Majewski M., Kasica N., Jakimiuk A., Podlasz P. (2018). Toxicity and cardiac effects of acute exposure to tryptophan metabolites on the kynurenine pathway in early developing zebrafish (*Danio rerio*) embryos. Toxicol. Appl. Pharmacol..

[79] Schmidt R., Strähle U., Scholpp S. (2013). Neurogenesis in zebrafish-from embryo to adult. Neural Dev..

[80] Wilson S.W., Ross L.S., Parrett T., Easter S.S. (1990). The development of a simple scaffold of axon tracts in the brain of the embryonic zebrafish, Brachydanio rerio. Development.

[81] Liu J., Kong W., Liu Y., Ma Q., Shao Q., Zeng L., Chao Y., Song X., Zhang J. (2023). Stage-related neurotoxicity of BPA in the development of zebrafish embryos. Toxics.

[82] Schwieler L., Trepci A., Krzyzanowski S., Hermansson S., Granqvist M., Piehl F., Venckunas T., Brazaitis M., Kamandulis S., Lindqvist D. (2020). A novel, robust method for quantification of multiple kynurenine pathway metabolites in the cerebrospinal fluid. Bioanalysis.

[83] Hu L.J., Li X.F., Hu J.Q., Ni X.J., Lu H.Y., Wang J.J., Huang X.N., Lin C.X., Shang D.W., Wen Y.G. (2017). A simple HPLC-MS/MS method for determination of tryptophan, kynurenine and kynurenic acid in human serum and its potential for monitoring antidepressant therapy. J. Anal. Toxicol..

[84] Sadik A., Somarribas Patterson L.F., Öztürk S., Mohapatra S.R., Panitz V., Secker P.F., Pfänder P., Loth S., Salem H., Prentzell M.T. (2020). IL4I1 Is a Metabolic immune checkpoint that activates the AHR and promotes tumor progression. Cell.

[85] Kaya B., Doñas C., Wuggenig P., Diaz O.E., Morales R.A., Melhem H., Hernández P.P., Kaymak T., Das S., Swiss IBD. Cohort Investigators (2020). Lysophosphatidic acid-mediated GPR35 signaling in CX3CR1^+^ macrophages regulates intestinal homeostasis. Cell Rep..

[86] Milligan G. (2018). G protein-coupled receptors not currently in the spotlight: Free fatty acid receptor 2 and GPR35. Br. J. Pharmacol..

[87] Seok S.H., Ma Z.X., Feltenberger J.B., Chen H., Chen H., Scarlett C., Lin Z., Satyshur K.A., Cortopassi M., Jefcoate C.R. (2018). Trace derivatives of kynurenine potently activate the aryl hydrocarbon receptor (AHR). J. Biol. Chem..

[88] Hahn B., Reneski C.H., Pocivavsek A., Schwarcz R. (2018). Prenatal kynurenine treatment in rats causes schizophrenia-like broad monitoring deficits in adulthood. Psychopharmacology.

[89] Buck S.A., Baratta A.M., Pocivavsek A. (2020). Exposure to elevated embryonic kynurenine in rats: Sex-dependent learning and memory impairments in adult offspring. Neurobiol. Learn. Mem..

[90] Takahashi T., Nowakowski R.S., Caviness V.S.J. (1995). Early ontogeny of the secondary proliferative population of the embryonic murine cerebral wall. J. Neurosci..

[91] Marszalek-Grabska M., Walczak K., Gawel K., Wicha-Komsta K., Wnorowska S., Wnorowski A., Turski W.A. (2021). Kynurenine emerges from the shadows—Current knowledge on its fate and function. Pharmacol Ther..

[92] Bubeníková-Valesová V., Horácek J., Vrajová M., Höschl C. (2008). Models of schizophrenia in humans and animals based on inhibition of NMDA receptors. Neurosci. Biobehav. Rev..

[93] Meltzer H.Y., Horiguchi M., Massey B.W. (2011). The role of serotonin in the NMDA receptor antagonist models of psychosis and cognitive impairment. Psychopharmacology.

[94] Gozzi A., Crestan V., Turrini G., Clemens M., Bifone A. (2010). Antagonism at serotonin 5-HT(2A) receptors modulates functional activity of frontohippocampal circuit. Psychopharmacology.

[95] Weisbrod A.S., Barry E.S., Graham A.M., Eklund M., Grunberg N.E. (2019). Decreased BDNF in female but not male rats after exposure to stress: A sex-sensitive rat model of stress?. Stress.

[96] Goodwill H.L., Manzano-Nieves G., Gallo M., Lee H.I., Oyerinde E., Serre T., Bath K.G. (2019). Early life stress leads to sex differences in development of depressive-like outcomes in a mouse model. Neuropsychopharmacology.

[97] Meier T.B., Drevets W.C., Teague T.K., Wurfel B.E., Mueller S.C., Bodurka J., Dantzer R., Savitz J. (2018). Kynurenic acid is reduced in females and oral contraceptive users: Implications for depression. Brain Behav. Immun..

[98] Mason M., Gullekson E.H. (1960). Estrogen-enzyme interactions: Inhibition and protection of kynurenine transaminase by the sulfate esters of diethylstilbestrol, estradiol, and estrone. J. Biol. Chem..

[99] Bao A.M., Swaab D.F. (2010). Sex differences in the brain, behavior, and neuropsychiatric disorders. Neuroscientist.

[100] Schwarcz R., Rassoulpour A., Wu H.Q., Medoff D., Tamminga C.A., Roberts R.C. (2001). Increased cortical kynurenate content in schizophrenia. Biol. Psychiatry.

[101] Wonodi I., Stine O.C., Sathyasaikumar K.V., Roberts R.C., Mitchell B.D., Hong L.E., Kajii Y., Thaker G.K., Schwarcz R. (2011). Downregulated kynurenine 3-monooxygenase gene expression and enzyme activity in schizophrenia and genetic association with schizophrenia endophenotypes. Arch. Gen. Psychiatry.

[102] Savitz J. (2020). The kynurenine pathway: A finger in every pie. Mol. Psychiatry.

[103] Prendergast B.J., Onishi K.G., Zucker I. (2014). Female mice liberated for inclusion in neuroscience and biomedical research. Neurosci. Biobehav. Rev..

[104] Palanza P., Parmigiani S. (2017). How does sex matter? Behavior, stress and animal models of neurobehavioral disorders. Neurosci. Biobehav. Rev..

[105] Jiang S., Lin L., Guan L., Wu Y. (2022). Selection of the male or female sex in chronic unpredictable mild stress-induced animal models of depression. Biomed. Res. Int..

[106] Jain S., Maltepe E., Lu M.M., Simon C., Bradfield C.A. (1998). Expression of ARNT, ARNT2, HIF1 alpha, HIF2 alpha and Ah receptor mRNAs in the developing mouse. Mech. Dev..

[107] Ganong A.H., Cotman C.W. (1986). Kynurenic acid and quinolinic acid act at N-methyl-D-aspartate receptors in the rat hippocampus. J. Pharmacol. Exp. Ther..

[108] Schwarcz R., Stone T.W. (2017). The kynurenine pathway and the brain: Challenges, controversies and promises. Neuropharmacology.

[109] Guidetti P., Schwarcz R. (1999). 3-Hydroxykynurenine potentiates quinolinate but not NMDA toxicity in the rat striatum. Eur. J. Neurosci..

[110] Guillemin G.J. (2012). Quinolinic acid, the inescapable neurotoxin. FEBS J..

[111] Venkatesan D., Iyer M., Narayanasamy A., Siva K., Vellingiri B. (2020). Kynurenine pathway in Parkinson’s disease—An update. eNeurologicalSci.

[112] Sathyasaikumar K.V., Tararina M., Wu H.Q., Neale S.A., Weisz F., Salt T.E., Schwarcz R. (2017). Xanthurenic acid formation from 3-hydroxykynurenine in the mammalian brain: Neurochemical characterization and physiological effects. Neuroscience.

[113] Lieberman H.R., Agarwal S., Fulgoni V.L. (2016). Tryptophan intake in the US adult population is not related to liver or kidney function but is associated with depression and sleep outcomes. J. Nutr..

[114] Castrogiovanni P., Musumeci G., Trovato F.M., Avola R., Magro G., Imbesi R. (2014). Effects of high-tryptophan diet on pre- and postnatal development in rats: A morphological study. Eur. J. Nutr..

[115] Musumeci G., Loreto C., Trovato F.M., Giunta S., Imbesi R., Castrogiovanni P. (2014). Serotonin (5HT) expression in rat pups treated with high-tryptophan diet during fetal and early postnatal development. Acta Histochem..

[116] Malaspina D., Corcoran C., Kleinhaus K.R., Perrin M.C., Fennig S., Nahon D., Friedlander Y., Harlap S. (2008). Acute maternal stress in pregnancy and schizophrenia in offspring: A cohort prospective study. BMC Psychiatry.

[117] Motlagh M.G., Katsovich L., Thompson N., Lin H., Kim Y.S., Scahill L., Lombroso P.J., King R.A., Peterson B.S., Leckman J.F. (2010). Severe psychosocial stress and heavy cigarette smoking during pregnancy: An examination of the pre- and perinatal risk factors associated with ADHD and Tourette syndrome. Eur. Child. Adolesc. Psychiatry.

[118] O’Donnell K.J., Glover V., Lahti J., Lahti M., Edgar R.D., Räikkönen K., O’Connor T.G. (2017). Maternal prenatal anxiety and child COMT genotype predict working memory and symptoms of ADHD. PLoS ONE.

[119] Schwabe L., Bohbot V.D., Wolf O.T. (2012). Prenatal stress changes learning strategies in adulthood. Hippocampus.

[120] Moura C.A., Cagni F.C., Costa L.R.F., Tiago P.R.F., Croyal M., Aguesse A., Reyes-Castro L.A., Zambrano E., Bolaños-Jiménez F., Gavioli E.C. (2022). Maternal stress during pregnancy in mice induces sex-dependent behavioral alterations in offspring along with impaired serotonin and kynurenine pathways of tryptophan metabolism. Dev. Neurosci..

[121] Fineberg A.M., Ellman L.M., Schaefer C.A., Maxwell S.D., Shen L., Chaudhury N.H., Cook A.L., Bresnahan M.A., Susser E.S., Brown A.S. (2016). Fetal exposure to maternal stress and risk for schizophrenia spectrum disorders among offspring: Differential influences of fetal sex. Psychiatry Res..

[122] Li J., Olsen J., Vestergaard M., Obel C. (2010). Attention-deficit/hyperactivity disorder in the offspring following prenatal maternal bereavement: A nationwide follow-up study in Denmark. Eur. Child Adolesc. Psychiatry.

[123] Zhu P., Hao J.H., Tao R.X., Huang K., Jiang X.M., Zhu Y.D., Tao F.B. (2015). Sex-specific and time-dependent effects of prenatal stress on the early behavioral symptoms of ADHD: A longitudinal study in China. Eur. Child Adolesc. Psychiatry.

[124] Van Lieshout R.J., Boylan K. (2010). Increased depressive symptoms in female but not male adolescents born at low birth weight in the offspring of a national cohort. Can. J. Psychiatry.

[125] Favaro A., Tenconi E., Degortes D., Manara R., Santonastaso P. (2015). Neural correlates of prenatal stress in young women. Psychol. Med..

[126] Iwata M., Ota K.T., Duman R.S. (2013). The inflammasome: Pathways linking psychological stress, depression, and systemic illnesses. Brain Behav. Immun..

[127] Rohleder N. (2019). Stress and inflammation: The need to address the gap in the transition between acute and chronic stress effects. Psychoneuroendocrinology.

[128] Takikawa O., Tagawa Y., Iwakura Y., Yoshida R., Truscott R.J. (1999). Interferon-gamma-dependent/independent expression of indoleamine 2,3-dioxygenase. Studies with interferon-gamma-knockout mice. Adv. Exp. Med. Biol..

[129] Popov A., Abdullah Z., Wickenhauser C., Saric T., Driesen J., Hanisch F.G., Domann E., Raven E.L., Dehus O., Hermann C. (2006). Indoleamine 2,3-dioxygenase-expressing dendritic cells form suppurative granulomas following *Listeria monocytogenes* infection. J. Clin. Investig..

[130] Maes M., Leonard B.E., Myint A.M., Kubera M., Verkerk R. (2011). The new “5-HT” hypothesis of depression: Cell-mediated immune activation induces indoleamine 2, 3-dioxygenase, which leads to lower plasma tryptophan and an increased synthesis of detrimental tryptophan catabolites (TRYCATs), both of which contribute to the onset of depression. Prog. Neuro-Psychopharmacol. Biol. Psychiatry.

[131] Erhardt S., Blennow K., Nordin C., Skogh E., Lindström L.H., Engberg G. (2001). Kynurenic acid levels are elevated in the cerebrospinal fluid of patients with schizophrenia. Neurosci. Lett..

[132] Kim Y.K., Jeon S.W. (2018). Neuroinflammation and the immune-kynurenine pathway in anxiety disorders. Curr. Neuropharmacol..

[133] Lovick T.A., Zangrossi H. (2021). Effect of estrous cycle on behavior of females in rodent tests of anxiety. Front. Psychiatry.

