# Supplementary material for: Developmental Exposure to Kynurenine Affects Zebrafish and Rat Behavior
Source: Cells. 2023 Sep 6;12(18):2224. doi: 10.3390/cells12182224 (PMC10526278; doi:10.3390/cells12182224)
Supplement: Supplementary file 1 [file cells-12-02224-s001.zip › cells-2579242-supplementary.pdf]

Supplementary materials

Fig. S1. Locomotor activity under the light-dark transition assay of 5-day-old larval zebrafish exposed to KYN from 1 to 72 (A–D), and from 49 to 72 (E–H) hpf. Distance traveled for 10 min by each larva during the light phase (A, E) and the dark phase (C, G); mean ± SD plotted in black, the median value displayed as a horizontal bar in blue. Median distance traveled for 10 min, calculated for each group during the light phase (B, F) and the dark phase (D, H). Number of subjects = 31–32 per group; *$p < 0.05$ vs. controls, non-parametric ANOVA. KYN – kynurenine. Note that the vertical axis is different on the left and right panels.

Fig. S2. Evidence for the formation of KYN metabolites by zebrafish larvae. Upper panel – the chromatogram shows the authentic KYN (standard), no significant chromatographic signal present; middle panel – the representative chromatogram shows recording obtained from medium + KYN 100 µM, incubation time 24 h; lower panel - the representative chromatogram shows recording obtained from medium + KYN 100 µM inhabited with zebrafish larvae (N= 20, 96-119 dpf), incubation time 24 h. Note that the detection conditions were optimally set for KYNA detection. All analyses were performed under identical chromatographic conditions. Green arrows indicate at least 3 peaks, representing 3 unidentified substances that were formed in the presence of KYN in the medium due to zebrafish larvae. Blue arrows indicate substances that have formed in the KYN medium without the presence of larvae. It is worth noting that there are 3 other peaks in the two lower panels, which represent spontaneously formed KYN derivatives. KYN-kynurenine.

Fig. S3. Identification of KYNA-like substance in zebrafish incubation medium by comparison of retention time of peaks in samples with authentic KYNA. Upper panel - the chromatogram shows the authentic KYNA (Sigma-Aldrich; standard), retention time 13.37 min; middle panel – the representative chromatogram shows recording obtained from medium + KYN 100 µM, incubation time 24 h; lower panel - the representative chromatogram shows recording obtained from medium + KYN 100 µM inhabited with zebrafish larvae (N=20, 96-119 dpf), incubation time 24 h. The vertical dashed line shows the retention time for the KYNA standard. No peak was found in the medium without KYN - data not shown. All analyses were performed under identical chromatographic conditions. KYN-kynurenine, KYNA-kynurenic acid.

Fig. S4. Identification of KYNA-like substance in zebrafish incubation medium by addition of an internal standard to the original sample. Upper panel - the chromatogram shows the authentic KYNA (Sigma-Aldrich; standard); middle panel – the representative chromatogram shows recording obtained from medium + KYN 100 µM inhabited with zebrafish larvae (N=20, 96-119 dpf), incubation time 24 h; lower panel - the chromatogram shows recording obtained from medium + KYN 100 µM inhabited with zebrafish larvae (N=20, 96-119 dpf), incubation time 24 h; after incubation samples were added with authentic KYNA (standard). Both lower panels show records derived from the same colony of larvae, yet the vertical scale is different on the two panels. The vertical dashed line shows the retention time for the KYNA standard. KYN-kynurenine, KYNA-kynurenic acid.

Fig. S5. Effect of KYN administration during ED 1–7 on the body mass gain of male (A) and female (B) rats. Data are presented as a mean ± standard error of the mean (SEM), number of subjects = 30–

40 per group, circles – controls, squares – KYN-treated rats. *$p < 0.05$ vs. respective controls. ED – embryonic day, PND – postnatal day.

Fig. S6. Effect of KYN administration during ED 1–7 on the spontaneous and stimulated locomotor activity of adult male rats. Data are presented as a mean ± SEM, number of subjects = 9 per group, circles – controls, squares – KYN-treated rats. *$p < 0.05$ vs. respective controls. ED – embryonic day.

Fig. S7. Effect of KYN administration during ED 1–7 on the spontaneous and stimulated locomotor activity of adult female rats. Data are presented as a mean ± SEM, number of subjects = 9 per group, circles – controls, squares – KYN-treated rats. *$p < 0.05$ vs. respective controls. ED – embryonic day.

Fig. S8. Effect of KYN administration during ED 1–7 on the anxiety-like behavior of adult rats, estimated in the elevated plus-maze test. Number of entries into the open arms (A, B), time spent by rats in the open arms (C, D), number of total entries into the both open and closed arms, as a read out of locomotor activity (E, F). Data are presented as a mean ± SEM, number of subjects = 10 per group, *$p < 0.05$ vs. respective controls. ED – embryonic day, KYN – kynurenine.

Fig. S9. Effect of KYN administration during ED 1–7 on the reversal learning of adult rats. Escape latency (A, B), errors committed (C, D), entries into the previous escape location (E, F). Data are presented as a mean ± SEM, number of subjects = 10 per group, *$p < 0.05$ vs. respective controls. ED – embryonic day, KYN – kynurenine.

Fig. S10. Effect of KYN administration during ED 1–7 on the associative learning of adult rats. Training of the fear conditioning test (A, B), contextual fear conditioning test (C, D), cue fear conditioning test (E, F). Data are presented as a mean ± SEM, number of subjects = 10 per group, *$p < 0.05$ vs. respective controls. ED – embryonic day, KYN – kynurenine.

Table S1. Effect of KYN administration during ED 1–7 on the level of tryptophan, KYN and KYNA in mother's plasma

Table S2. Effect of KYN administration during ED 1–7 on the level of tryptophan, KYN and KYNA in male offspring's plasma

Table S3. Effect of KYN administration during ED 1–7 on the level of tryptophan, KYN and KYNA in female offspring's plasma

Table S4. Effect of KYN administration during ED 1–7 on anxiety-like behavior and memory processes of adult male rats

Table S5. Effect of KYN administration during ED 1–7 on anxiety-like behavior and memory processes of adult female rats

Fig. S1.

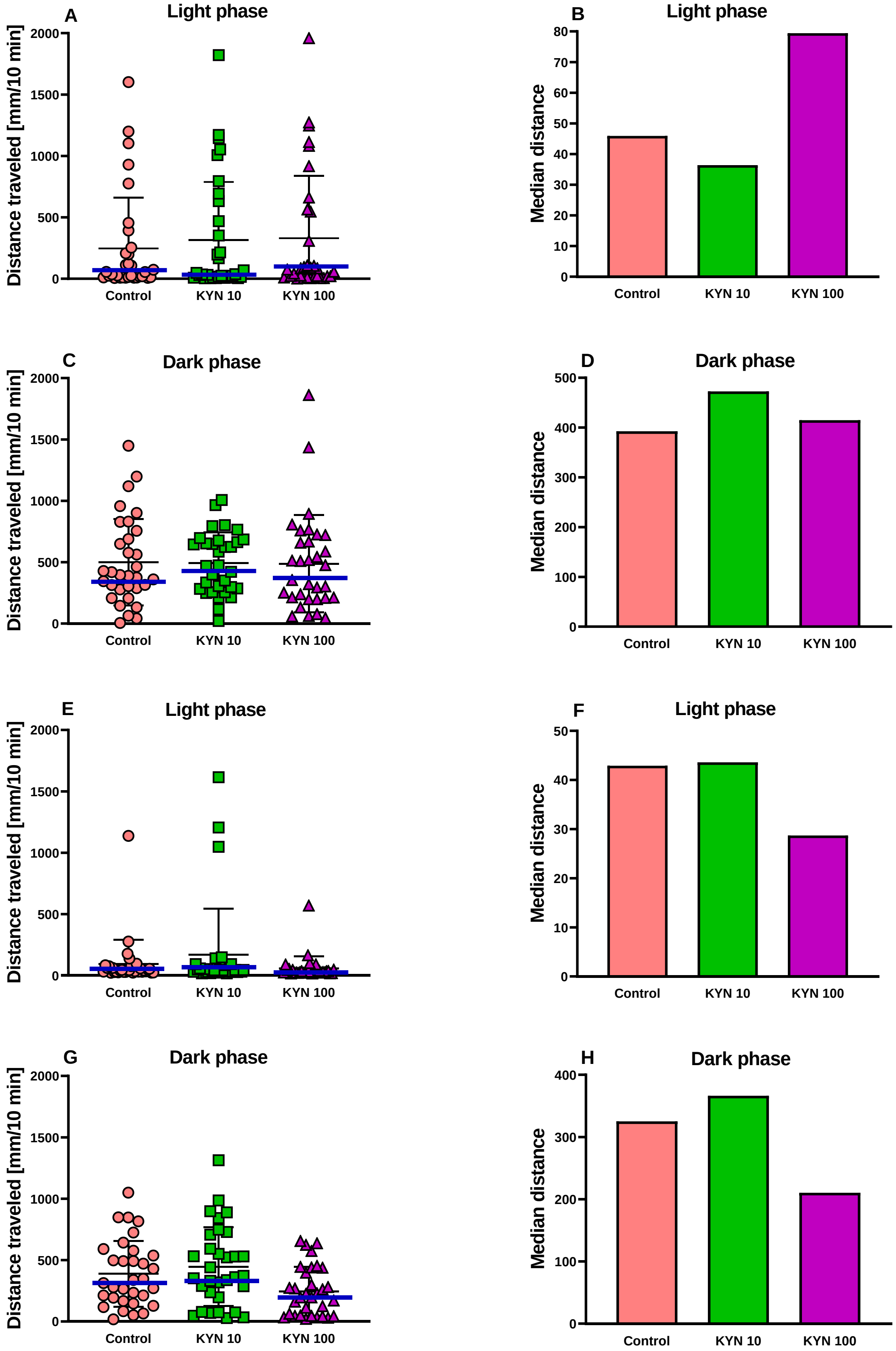

A
Light phase
Distance traveled [mm/10 min]
2000
1500
1000
500
0
Control
KYN 10
KYN 100
B
Light phase
Median distance
80
70
60
50
40
30
20
10
0
Control
KYN 10
KYN 100
C
Dark phase
Distance traveled [mm/10 min]
2000
1500
1000
500
0
Control
KYN 10
KYN 100
D
Dark phase
Median distance
500
400
300
200
100
0
Control
KYN 10
KYN 100
E
Light phase
Distance traveled [mm/10 min]
2000
1500
1000
500
0
Control
KYN 10
KYN 100
F
Light phase
Median distance
50
40
30
20
10
0
Control
KYN 10
KYN 100
G
Dark phase
Distance traveled [mm/10 min]
2000
1500
1000
500
0
Control
KYN 10
KYN 100
H
Dark phase
Median distance
400
300
200
100
0
Control
KYN 10
KYN 100

Fig. S2.

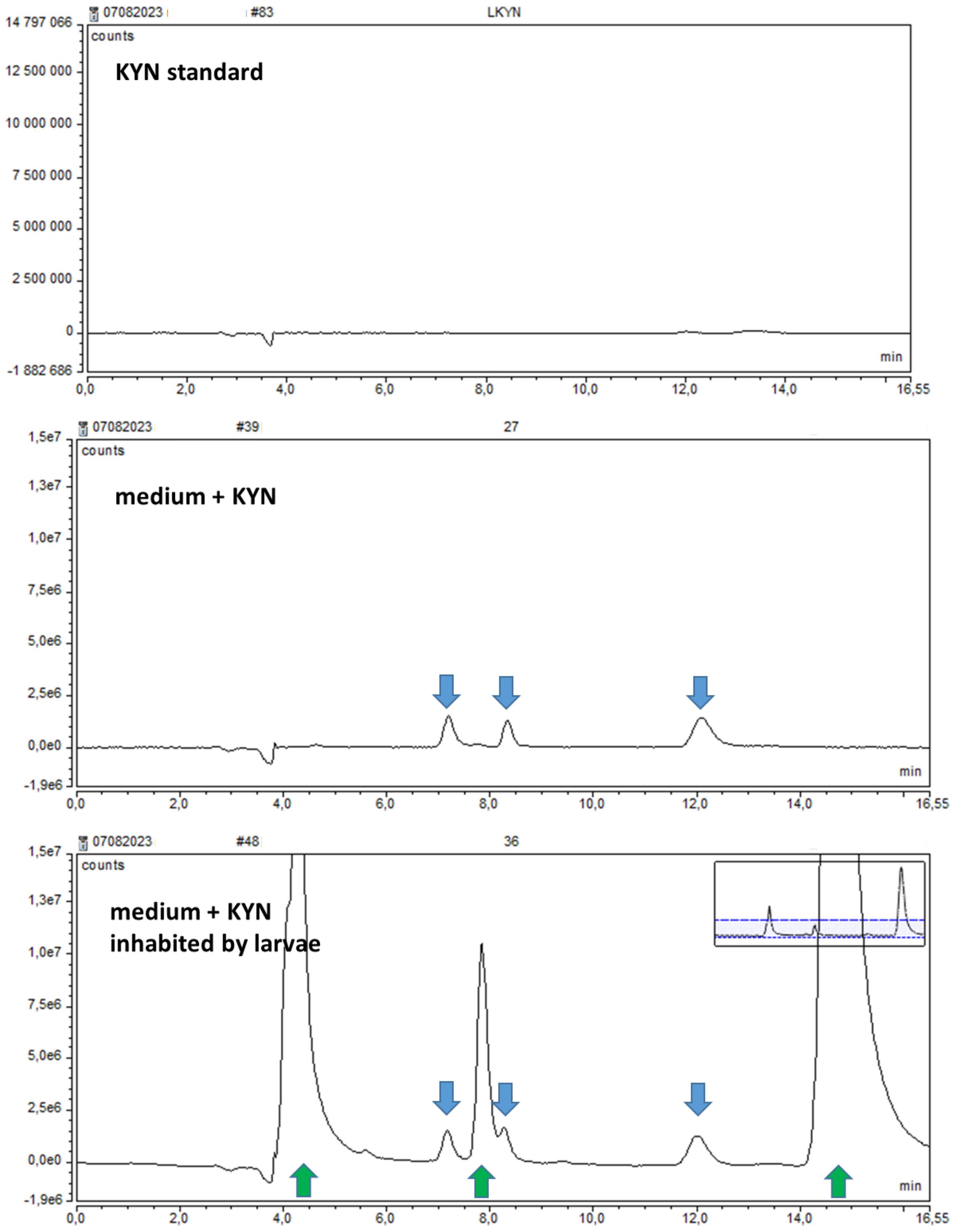
07082023
#83
LKYN
counts
KYN standard
14 797 066
12 500 000
10 000 000
7 500 000
5 000 000
2 500 000
0
-1 882 686
min
0,0
2,0
4,0
6,0
8,0
10,0
12,0
14,0
16,55
07082023
#39
27
counts
medium + KYN
1,5e7
1,3e7
1,0e7
7,5e6
5,0e6
2,5e6
0,0e0
-1,9e6
min
07082023
#48
36
counts
medium + KYN
inhabited by larvae
min

Fig. S3.

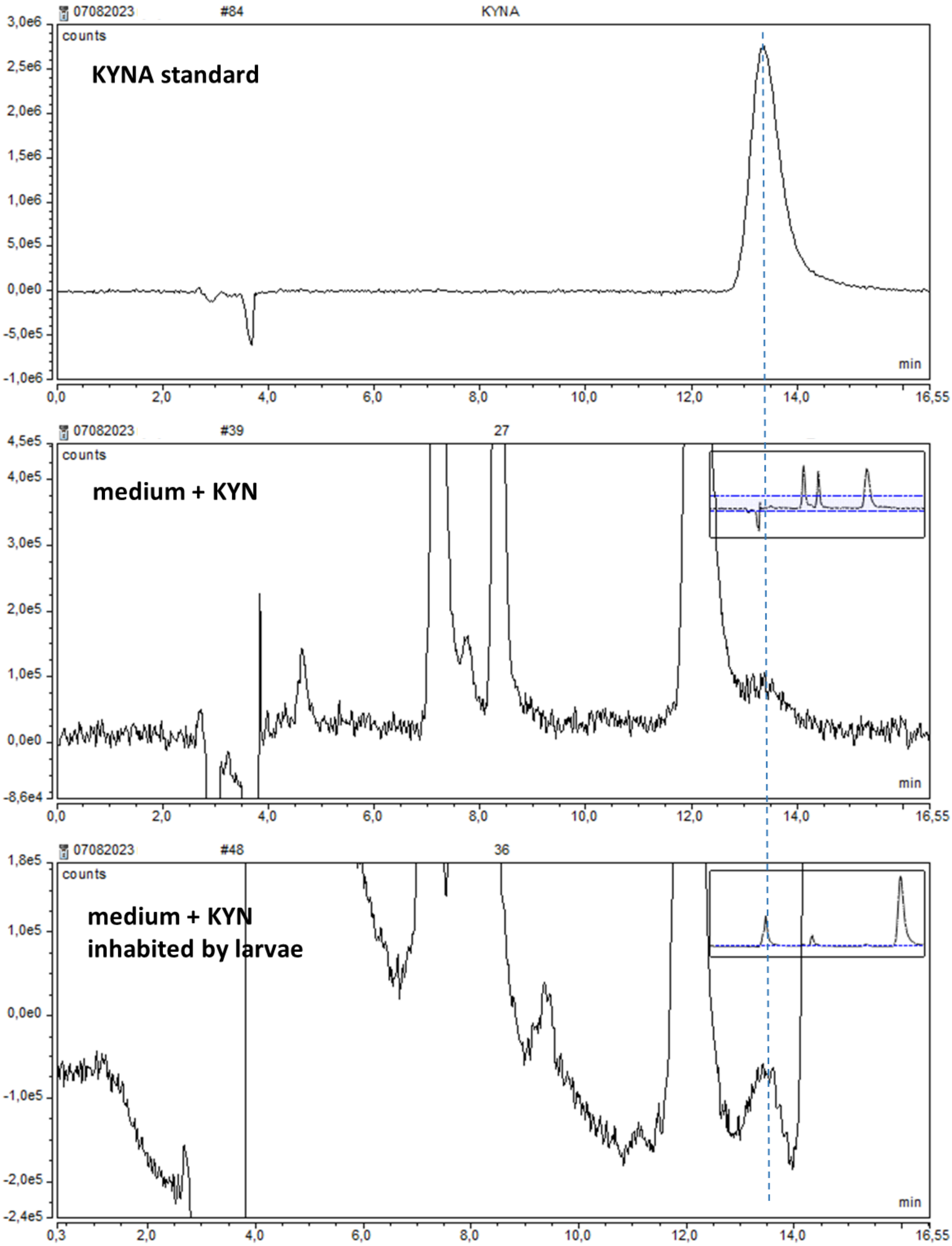

07082023
#84
KYNA
counts
KYNA standard
3,0e6
2,5e6
2,0e6
1,5e6
1,0e6
5,0e5
0,0e0
-5,0e5
-1,0e6
min
0,0
2,0
4,0
6,0
8,0
10,0
12,0
14,0
16,55
07082023
#39
27
counts
medium + KYN
4,5e5
4,0e5
3,0e5
2,0e5
1,0e5
0,0e0
-8,6e4
min
07082023
#48
36
counts
medium + KYN
inhabited by larvae
1,8e5
1,0e5
0,0e0
-1,0e5
-2,0e5
-2,4e5
min
0,3

Fig. S4.

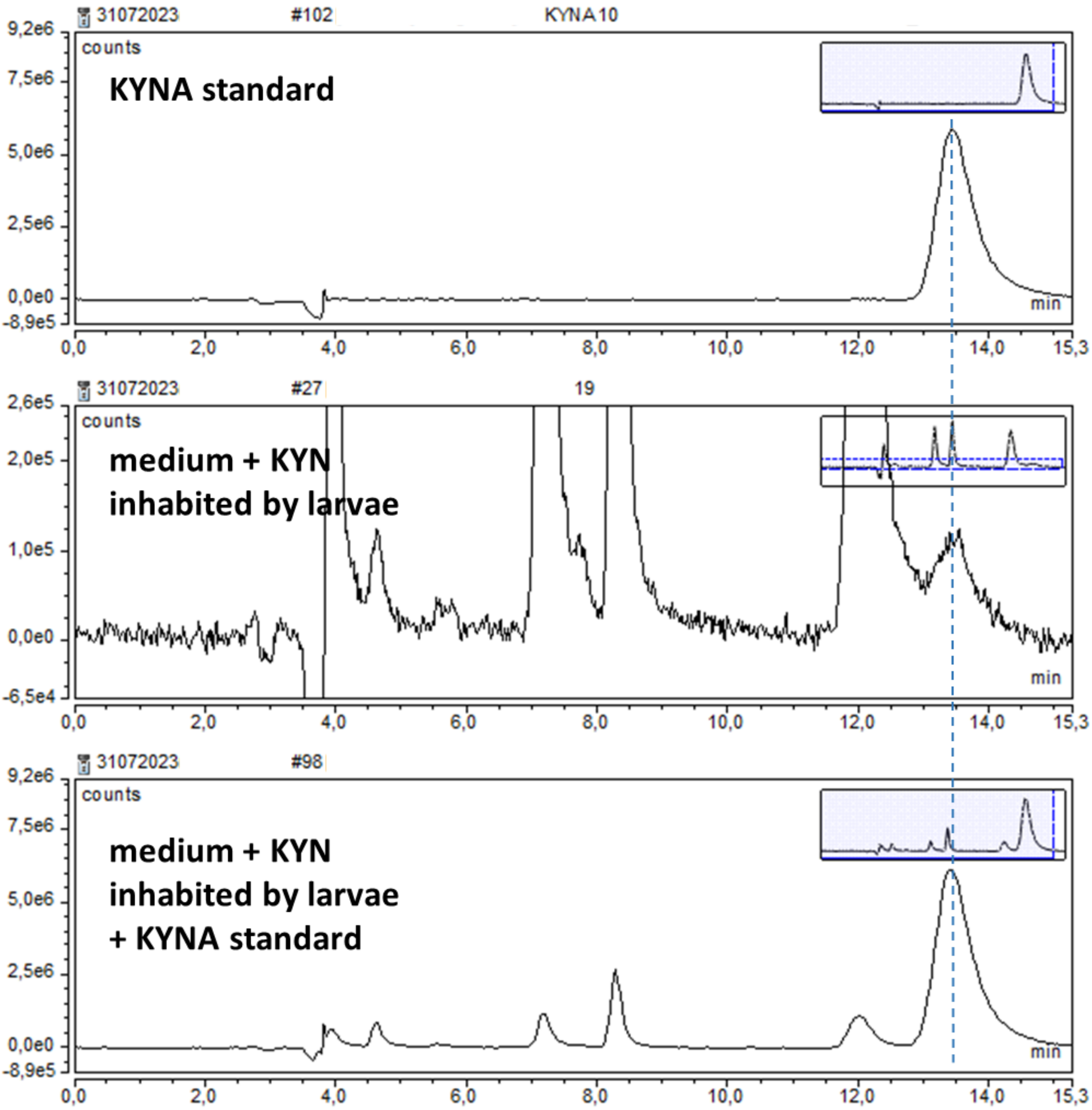
31072023
#102
KYNA 10
counts
KYNA standard
9,2e6
7,5e6
5,0e6
2,5e6
0,0e0
-8,9e5
min
0,0
2,0
4,0
6,0
8,0
10,0
12,0
14,0
15,3
31072023
#27
19
counts
medium + KYN
inhabited by larvae
2,6e5
2,0e5
1,0e5
0,0e0
-6,5e4
min
31072023
#98
counts
medium + KYN
inhabited by larvae
+ KYNA standard
9,2e6
7,5e6
5,0e6
2,5e6
0,0e0
-8,9e5
min

Fig. S5.

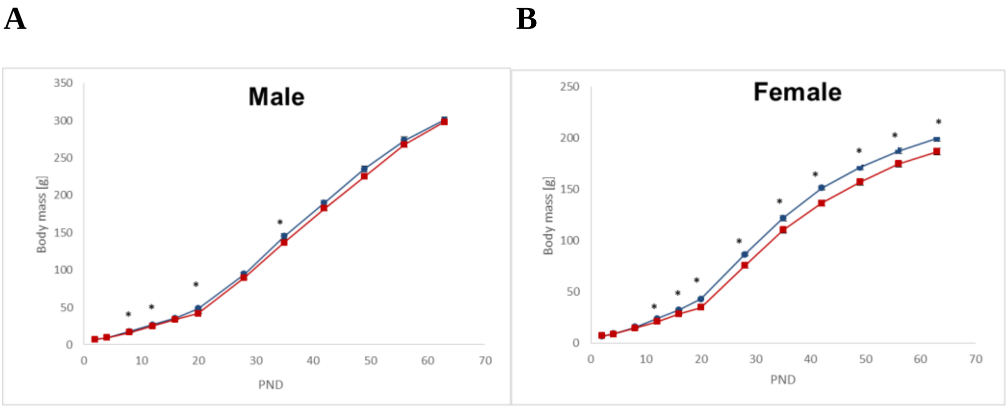

Fig. S6.

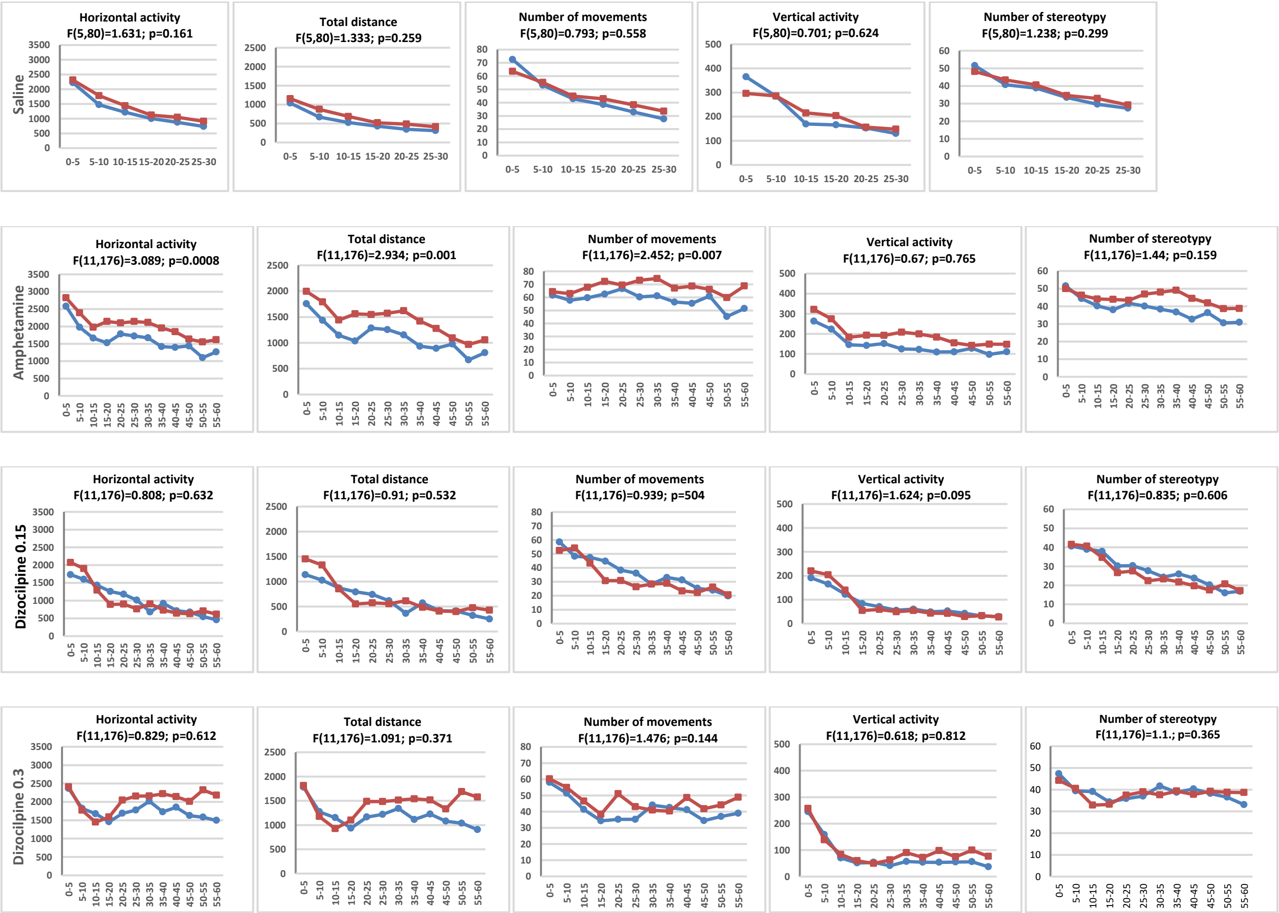
Saline
Horizontal activity
F(5,80)=1.631; p=0.161
Total distance
F(5,80)=1.333; p=0.259
Number of movements
F(5,80)=0.793; p=0.558
Vertical activity
F(5,80)=0.701; p=0.624
Number of stereotypy
F(5,80)=1.238; p=0.299
Amphetamine
Horizontal activity
F(11,176)=3.089; p=0.0008
Total distance
F(11,176)=2.934; p=0.001
Number of movements
F(11,176)=2.452; p=0.007
Vertical activity
F(11,176)=0.67; p=0.765
Number of stereotypy
F(11,176)=1.44; p=0.159
Dizocilpine 0.15
Horizontal activity
F(11,176)=0.808; p=0.632
Total distance
F(11,176)=0.91; p=0.532
Number of movements
F(11,176)=0.939; p=504
Vertical activity
F(11,176)=1.624; p=0.095
Number of stereotypy
F(11,176)=0.835; p=0.606
Dizocilpine 0.3
Horizontal activity
F(11,176)=0.829; p=0.612
Total distance
F(11,176)=1.091; p=0.371
Number of movements
F(11,176)=1.476; p=0.144
Vertical activity
F(11,176)=0.618; p=0.812
Number of stereotypy
F(11,176)=1.1.; p=0.365

Fig. S7.

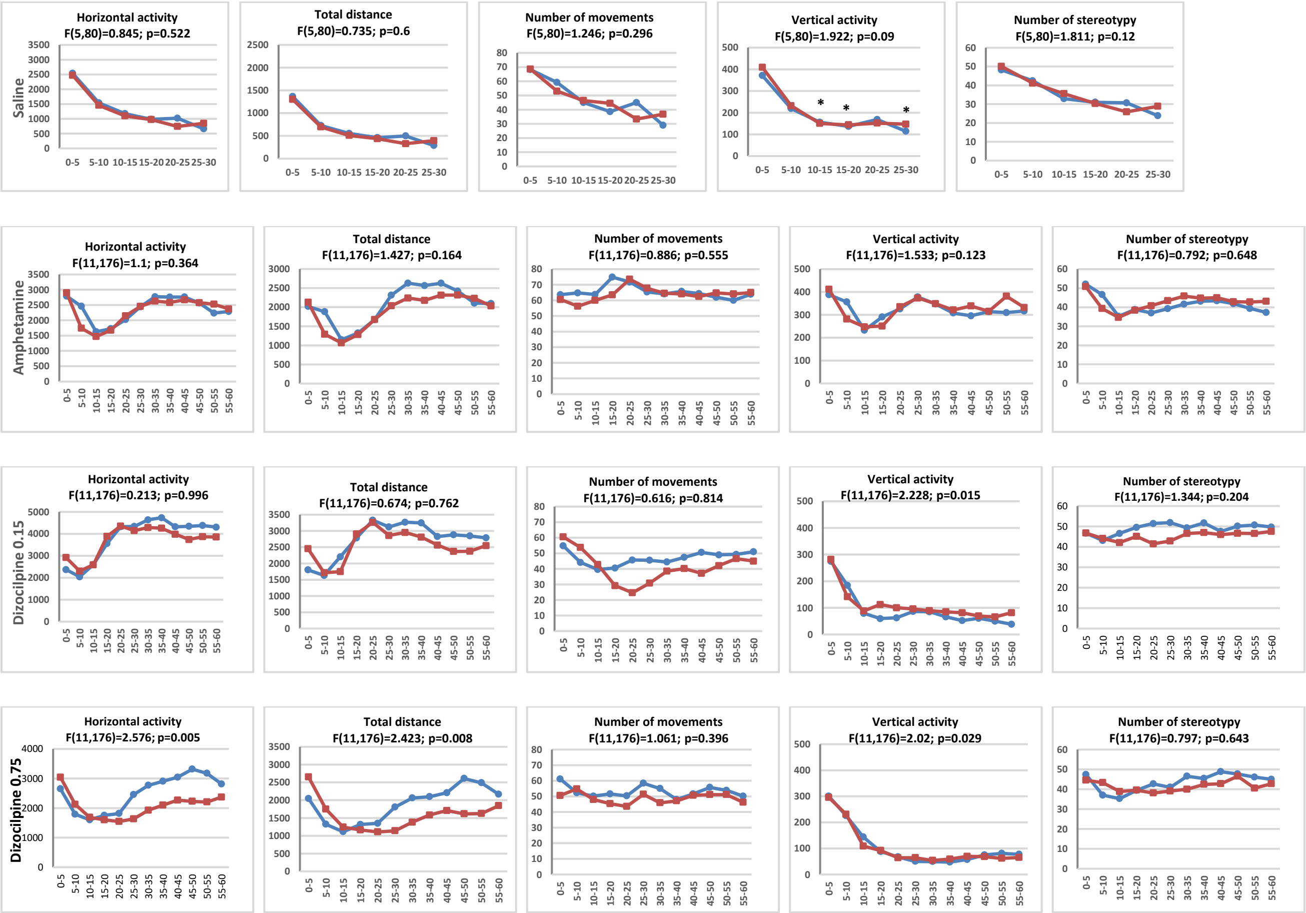
Saline
Horizontal activity
F(5,80)=0.845; p=0.522
Total distance
F(5,80)=0.735; p=0.6
Number of movements
F(5,80)=1.246; p=0.296
Vertical activity
F(5,80)=1.922; p=0.09
Number of stereotypy
F(5,80)=1.811; p=0.12
Amphetamine
Horizontal activity
F(11,176)=1.1; p=0.364
Total distance
F(11,176)=1.427; p=0.164
Number of movements
F(11,176)=0.886; p=0.555
Vertical activity
F(11,176)=1.533; p=0.123
Number of stereotypy
F(11,176)=0.792; p=0.648
Dizocilpine 0.15
Horizontal activity
F(11,176)=0.213; p=0.996
Total distance
F(11,176)=0.674; p=0.762
Number of movements
F(11,176)=0.616; p=0.814
Vertical activity
F(11,176)=2.228; p=0.015
Number of stereotypy
F(11,176)=1.344; p=0.204
Dizocilpine 0.75
Horizontal activity
F(11,176)=2.576; p=0.005
Total distance
F(11,176)=2.423; p=0.008
Number of movements
F(11,176)=1.061; p=0.396
Vertical activity
F(11,176)=2.02; p=0.029
Number of stereotypy
F(11,176)=0.797; p=0.643

Fig. S8.

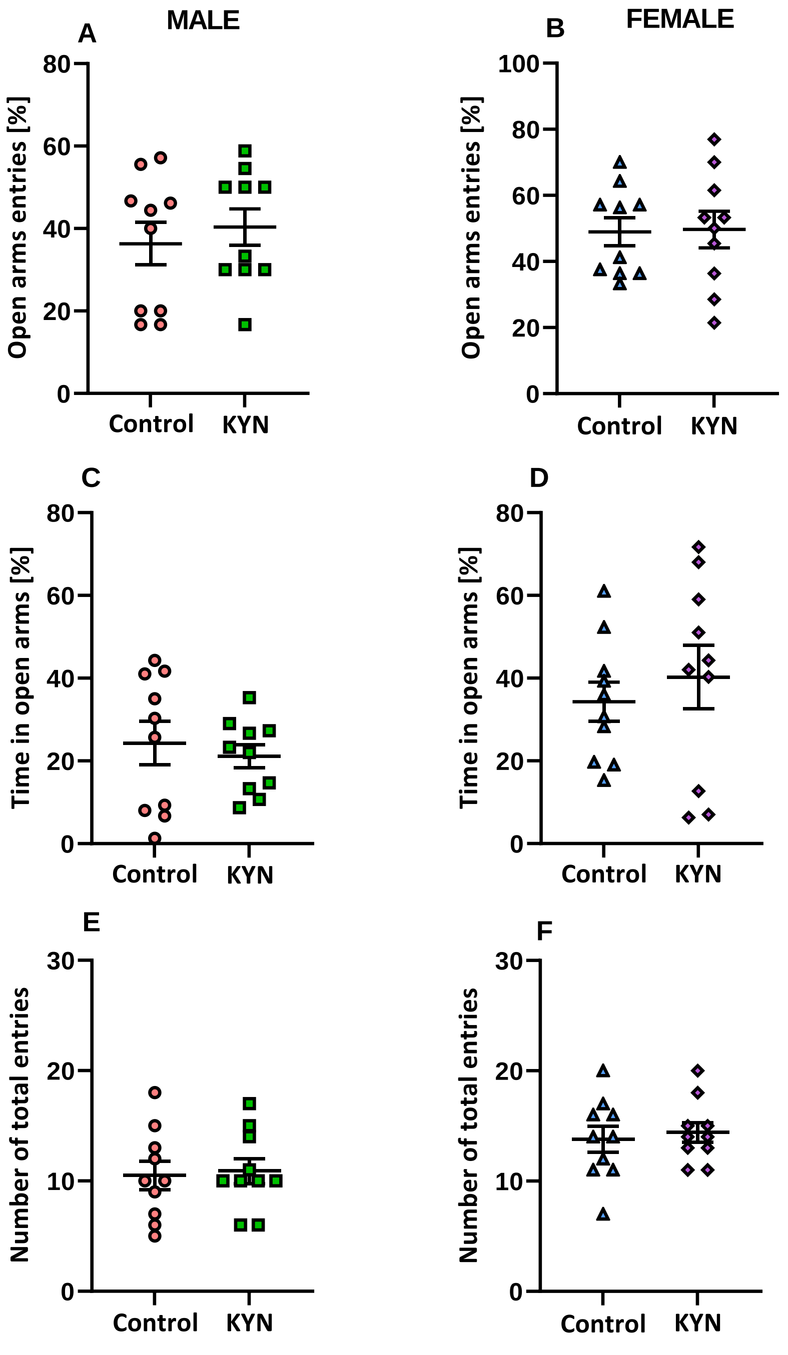
MALE
FEMALE
A
B
C
D
E
F
Open arms entries [%]
Time in open arms [%]
Number of total entries
Control
KYN
0
20
40
60
80
100
0
10
20
30

Fig. S9.

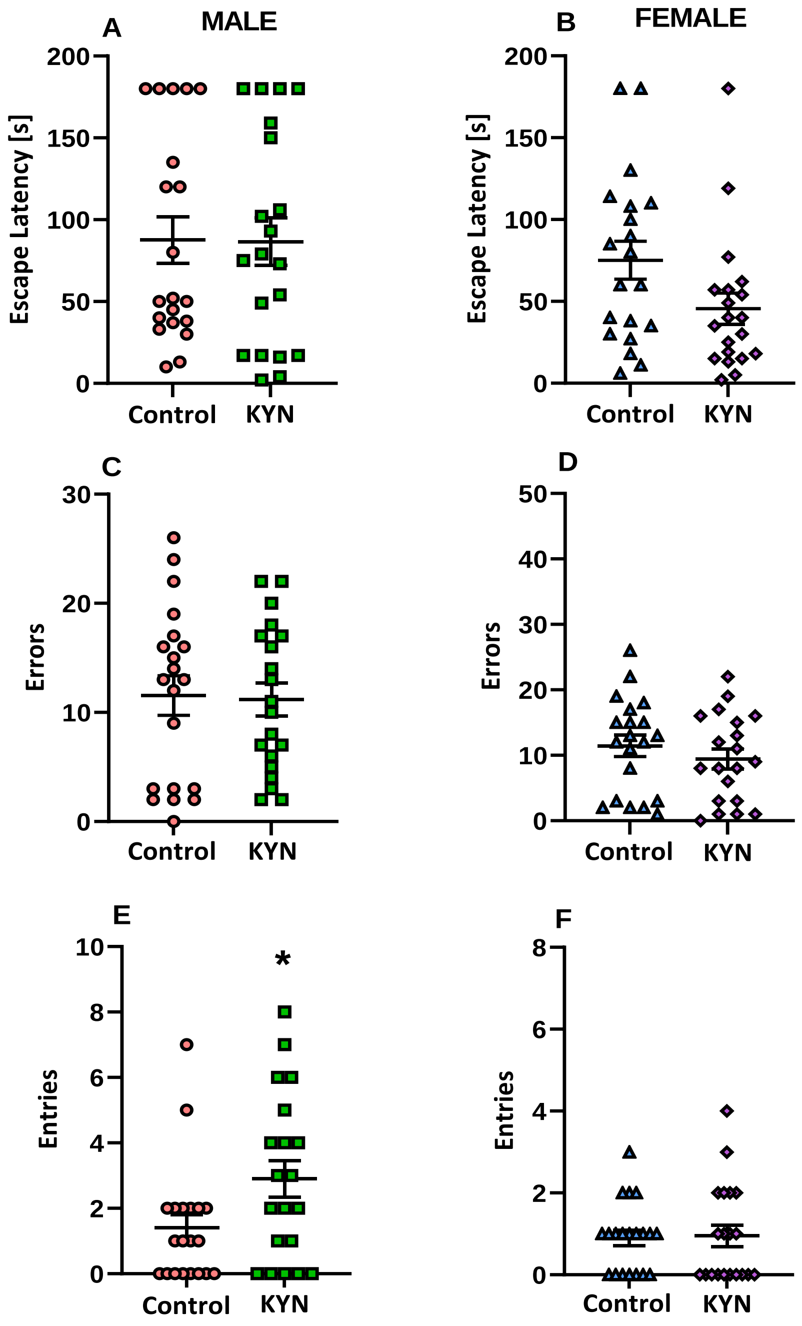
A
MALE
B
FEMALE
Escape Latency [s]
0
50
100
150
200
Control
KYN
C
D
Errors
0
10
20
30
40
50
E
F
Entries
0
2
4
6
8
10
*

Fig. S10.

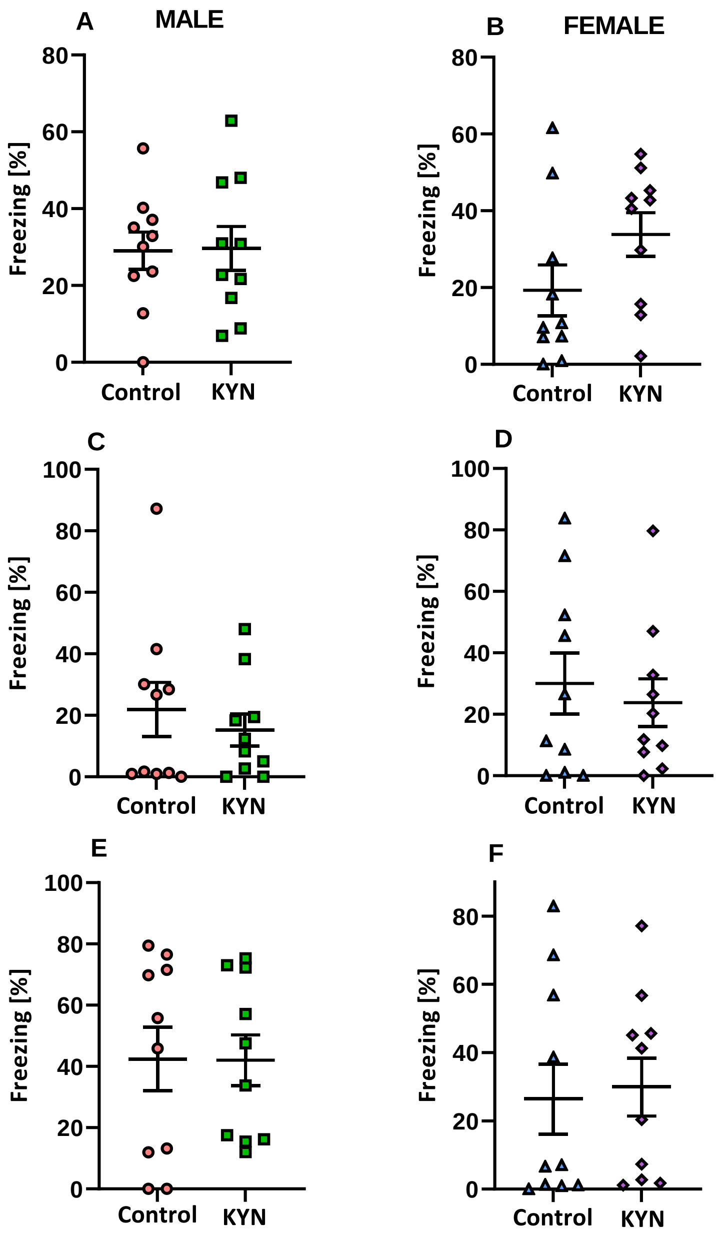

Table S1.

Compound [mM]	Control	KYN-treated	p <0.05 vs control
Tryptophan	56.44±10.52	125.35±12.07	YES
KYN	1.08±0.19	2.11±0.57	YES
KYNA	0.11±0.02	0.27±0.03	YES

Blood was collected on day 21 postpartum; data are presented as a mean±SEM; N=6 per group

Table S2.

Compound [mM]	Young		p <0.05 vs control	Adult		p <0.05 vs control
	Control	KYN-treated		Control	KYN-treated	
Tryptophan	98.40±4.36	85.97±2.28	YES	122.96±4.45	99.44±3.26	YES
KYN	1.19±0.13	2.30±0.40	YES	2.40±0.09	2.27±0.10	NO
KYNA	0.16±0.001	0.13±0.004	YES	0.05±0.003	0.05±0.005	NO

Data are presented as a mean±SEM; N=9-10 per group

Table S3.

Compound [mM]	Young		p <0.05 vs control	Adult		p <0.05 vs control
	Control	KYN-treated		Control	KYN-treated	
Tryptophan	85.63±6.96	90.41±2.69	NO	117.37±5.72	84.38±6.76	YES
KYN	1.16±0.14	1.69±0.13	YES	2.52±0.09	2.82±0.24	NO
KYNA	0.14±0.02	0.13±0.004	NO	0.07±0.005	0.09±0.012	NO

Data are presented as a mean±SEM; N=9-10 per group

Table S4.

Elevated plus-maze test	Open arms entries	=
	Time in open arms	=
	Number of total entries	=
Barnes maze test	Escape latency	=
	Errors	=
	Entries	↑
Contextual and cued fear conditioning	Training	=
	Contextual fear conditioning test	=
	Cue fear conditioning test	=

= no effect, ↑ increase

Table S5.

Elevated plus-maze test	Open arms entries	=
	Time in open arms	=
	Number of total entries	=
Barnes maze test	Escape latency	=
	Errors	=
	Entries	=
Contextual and cued fear conditioning	Training	=
	Contextual fear conditioning test	=
	Cue fear conditioning test	=

= no effect
